# Identification of Endoplasmic Reticulum Stress-Related Genes in Osteoporosis Pathogenesis

**DOI:** 10.1155/mi/6726771

**Published:** 2025-08-30

**Authors:** Yiren Zhu, Xiu Yang, Yunan Lu, Jiayu He, Bo Liu, Yongfa Zhang, Zhengchao Zhang

**Affiliations:** ^1^Shengli Clinical Medical College of Fujian Medical University, Fuzhou 350001, Fujian, China; ^2^Fuzong Clinical Medical College of Fujian Medical University, Fuzhou 350001, Fujian, China; ^3^Department of Paediatric Orthopaedics, Fuzhou Second Hospital, Fuzhou, Fujian Province 350007, China; ^4^Fuzhou University Affiliated Provincial Hospital, Fuzhou 350001, Fujian, China; ^5^Department of Emergency Trauma Surgery, Fujian Provincial Hospital, Fuzhou 350001, Fujian, China; ^6^Fujian Trauma Medicine Center, Fuzhou 350001, Fujian, China; ^7^Fujian Key Laboratory of Emergency Medicine, Fuzhou 350001, Fujian, China

**Keywords:** bioinformatics, diagnostic model, endoplasmic reticulum stress, immune infiltration, osteoporosis, regulatory networks

## Abstract

**Background:** Osteoporosis is a prevalent metabolic bone disorder with complex molecular underpinnings. Emerging evidence implicates endoplasmic reticulum stress (ERS) in its pathogenesis; however, systematic exploration of ERS-related genes (ERSRGs) remains limited. This study aimed to identify ERS-related differentially expressed genes (ERSRDEGs) in osteoporosis, construct a diagnostic model, and elucidate associated molecular mechanisms.

**Methods:** Three osteoporosis datasets (GSE56815, GSE230665, and GSE7429) were integrated after batch effect correction and normalization. ERSRGs were curated from GeneCards, and ERSRDEGs were identified by intersecting co-differentially expressed genes (Co-DEGs) across datasets. Functional enrichment (gene set enrichment analysis [GSEA], gene set variation analysis [GSVA], Gene Ontology [GO], and Kyoto Encyclopedia of Genes and Genomes [KEGG]) and immune infiltration analyses were performed. Diagnostic models were developed using support vector machine (SVM) and least absolute shrinkage and selection operator (LASSO) regression, validated via receiver operating characteristic (ROC) curves, nomograms, and decision curve analysis. Experimental validation included immunohistochemistry and quantitative reverse transcription polymerase chain reaction (qRT-PCR) in ovariectomized (OVX) mice. Regulatory networks (TF-miRNA-RBP-drug) and protein structure predictions were generated using bioinformatic tools.

**Results:** Fifty six ERSRDEGs were identified, enriched in apoptosis, autophagy, and cytokine signaling pathways. A diagnostic model comprising seven genes (CYB5R4, RAB1B, UFSP2, RNF13, SERP1, CES2, and C1QBP) demonstrated high accuracy (area under the curve (AUC) > 0.9) in both training and validation datasets. Immune infiltration analysis revealed distinct patterns of activated B cells, CD8^+^ T cells, and macrophages between high- and low-risk groups. Regulatory networks highlighted interactions with 52 transcription factors (TFs), 42 miRNAs, and 27 therapeutic compounds. Experimental validation in OVX mice confirmed upregulated expression of C1QBP, CYB5R4, RAB1B, and UFSP2 at protein/mRNA levels, aligning with bioinformatic predictions.

**Conclusions:** This study establishes ERSRDEGs as critical players in osteoporosis pathogenesis and provides a clinically translatable seven-gene diagnostic model for early osteoporosis detection. The integration of multiomics analyses uncovered key pathways, immune dynamics, and regulatory networks, while experimental validation reinforced the role of specific ERSRGs. These findings provide novel insights into ERS-mediated mechanisms and therapeutic targets for osteoporosis management.

## 1. Introduction

In identification of endoplasmic reticulum stress-related genes (ERSRGs), Osteoporosis Pathogenesis International Osteoporosis Foundation reports that nearly 200 million women worldwide are affected by osteoporosis, resulting in approximately eight million fractures every year, equivalent to one osteoporotic fracture occurring every 3 seconds [1]. The risk of mortality increases considerably in the first year following a fracture, with some studies indicating a 20% mortality rate within 12 months after a hip fracture; however, recent research suggests a lower figure of approximately 9% [2].The current diagnostic approach primarily relies on dual-energy X-ray absorptiometry (DXA) to measure bone mineral density (BMD). However, these methods have limitations in accurately predicting fractures and fail to capture the intricate biological mechanisms involved in bone loss [3].

Important markers in the study of osteoporosis, such as serum osteocalcin, bone-specific alkaline phosphatase, and C-terminal telopeptide of Type I collagen (CTX), have been identified as valuable tools for determining the onset and potential outcomes of the condition [4]. Various methods, including enzyme-linked immunosorbent assay (ELISA), quantitative reverse transcription polymerase chain reaction (qRT-PCR), and bioinformatics analysis, are utilized to identify osteoporosis biomarkers [5].

The endoplasmic reticulum stress (ERS) response is now acknowledged as a significant factor in a range of diseases, such as neurodegenerative disorders and cancer [6]. ERS plays a crucial role in the differentiation and function of osteoblasts and osteoclasts in bone biology, suggesting its potential contribution to the pathogenesis of osteoporosis. Recent research has indicated that the manipulation of genes associated with ERS may impact the density and strength of bones, offering promising avenues for the treatment of osteoporosis [7]. However, the full extent of ERS's involvement in osteoporosis still necessitates further investigation.

This study aims to bridge this gap by employing bioinformatics tools to investigate gene expression patterns associated with ERS in osteoporosis. We analyzed and processed gene expression datasets from the Gene Expression Omnibus (GEO) utilizing robust statistical approaches, developed diagnostic models using differentially expressed genes (DEGs) associated with ERS, and experimentally validated key candidate genes in an ovariectomy-induced mouse model. Through integrated approaches including immunohistochemistry and qRT-PCR confirming the dysregulation of critical ERSRGs at both transcriptional and translational levels. Furthermore, the study investigates the patterns of immune infiltration in the disease context and predicts crucial regulatory networks that could serve as promising therapeutic targets.

## 2. Materials and Methods

### 2.1. Data Download

Three datasets related to osteoporosis, specifically GSE56815 [8], GSE230665 [9], and GSE7429 [10], were extracted from the GEO database using the R package GEO query [11]. Further detailed information can be found in Supporting Information 1: Table S1. All samples were thoroughly evaluated in the analysis.

A study on ERSRGs [12] was carried out by utilizing the GeneCards database. The search term “Endoplasmic reticulum stress” was employed, and only ERSRGs categorized as “Protein Coding” were included.

The R package “sva” [13] was used to remove batch effects from the datasets GSE56815 and GSE230665 to obtain the integrated GEO dataset. Following this, the integrated combined dataset underwent standardization using the R package limma [14], with probes being annotated before standardization and normalization. Principal component analysis (PCA) [15] was conducted on the expression matrix to evaluate the impact of batch effect removal.

### 2.2. ERS-Related DEGs (ERSRDEGs)

The R limma package was used to compare osteoporosis samples with control samples and the gene expression variance was calculated within the combined GEO dataset.

GSE7429 was then differentially analyzed using the same procedure and screening criteria as the GEO combined dataset. Co-DEGs were obtained by intersecting DEGs in the GEO combined dataset with DEGs in GSE7429.

The intersection of Co-DEGs and ERSRGs was used to create a Venn diagram to identify ERSRDEGs.

### 2.3. Gene Set Enrichment Analysis (GSEA)

The GSEA method [16] is employed to evaluate the pattern of gene distribution in a defined set of genes in a gene table organized by phenotypic correlation. In this study, genes in the integrated GEO dataset (pooled dataset) were initially sorted by logFC value, followed by GSEA using the R package clusterProfiler [17]. The significance criterion for GSEA settings consisted of the seed (2022), the number of iterations (1000), the minimum gene count per group (10), and the maximum gene count (500). The gene collection c2.cp.all.v2022.1.Hs.symbols.gmt (All Canonical Pathways; 3050) was obtained from the Molecular Signatures Database (MSigDB) [18] for GSEA analysis. GSEA set at *p* < 0.05.

### 2.4. Gene Set Variation Analysis (GSVA)

GSVA [19] was employed to quantitatively evaluate gene set enrichment results in the context of a microarray-based transcriptome. In examining datasets related to osteoporosis, GSVA was conducted on all genes using the gene set c2.cp.all.v2022.1.Hs.symbols.gmt from the MSigDB. GSVA set at *p* < 0.05.

### 2.5. Gene Ontology (GO) and Kyoto Encyclopedia of Genes and Genomes (KEGG)

The analysis of GO [20] is frequently used in broad functional enrichment investigations, encompassing the study of biological process (BP), cellular component (CC), and molecular function (MF). The KEGG [21] is a widely accessed database containing information on genetic material, biological pathways, diseases, and drugs. Using the R package clusterProfiler, ERSRDEGs were subjected to enrichment analysis based on both GO and KEGG, with a significance threshold of *p* < 0.05 for inclusion. To visualize the results of the KEGG enrichment analysis, the R package Pathview [22] was employed.

### 2.6. Construction of Osteoporosis Diagnostic Model

Using the combined GEO dataset, a model was developed for the diagnosis of osteoporosis. Logistic regression was performed on the ERSRDEGs to investigate the correlation between independent variables and a binary dependent variable, distinguishing osteoporosis samples from control samples. By setting a significance level of *p* < 0.1, the ERSRDEGs were identified and incorporated into the logistic regression model.

Afterward, we employed the Mann–Whitney *U* test to assess the ERSRG expression levels across the combined dataset and the GSE7429 dataset. Differential expression of genes related to ERS was compared based on expression differences and visually represented in group comparison plots. Subsequently, only the differentially expressed ERSRGs that showed significant differences between the osteoporosis and normal groups in both datasets were retained for further analysis.

Next, the support vector machine (SVM) algorithm [23] was utilized to establish an SVM model for the ERSRDEGs identified in the group comparison plot.

At the same time, we utilized the R package glmnet [24] to perform least absolute shrinkage and selection operator (LASSO) analysis on the ERSRDEGs maintained in the group comparison chart. LASSO regression is rooted in linear regression, incorporating a penalty term (lambda × absolutevalue of the coefficient) to mitigate overfitting and enhance model generalization. The outcomes of the LASSO regression analysis can be displayed visually using diagnostic model plots and variable trajectory graphs.

The identified ERSRDEGs in both the LASSO model and SVM were compared using a Venn diagram to establish the model genes. Next, the coefficients of these genes from the LASSO model and the dataset expression were merged to create the diagnostic model for osteoporosis, outlined as follows.

### 2.7. Validation of Osteoporosis Diagnostic Model

Using logistic regression analysis outcomes in the R package rms, a nomogram [25] is generated as a graphical representation in a rectangular coordinate system. The R package ggDCA is capable of producing a DCA graph for the model genes identified in the combined dataset. This graph provides valuable information regarding the clinical significance and overall advantages of the predictive model across different probability thresholds [26].

Following this, subsequently, the R package pROC was used to generate receiver operating characteristic (ROC) curves and calculate area under the curve (AUC) values, evaluating the diagnostic performance of the LASSO risk score in osteoporosis.

Using the formula for computing the risk score in the osteoporosis diagnosticmodel, the dataset GSE7429 underwent computation of the risk score. Utilizing the R package (pROC), an ROC curve was generated to calculate the AUC for the dataset. The samples from the combined dataset were then divided into high-risk and low-risk groups using the median risk score from the diagnostic model for osteoporosis.

### 2.8. Immune Infiltration Analysis

The evaluation of gene sets for individual samples (ssGSEA) [27] evaluates the proportion of infiltrating immune cells. Initially, the different types of infiltrating immune cells were identified, the enrichment score derived from ssGSEA was utilized to indicate the proportion of immune cell infiltration in each individual sample. In order to compare the level of immune cell infiltration between samples from high-risk and low-risk groups in the merged dataset, group comparison diagrams were utilized, with the Spearman statistical method employed for computation.

CIBERSORT [28] utilizes linear support vector regression to analyze the transcriptome expression matrix, assessing the composition and presence of immune cells within mixed cell populations. Subsequent analysis involves using the ggplot2 R package to generate comparative charts that highlight differences in LM22 immune cell expressions between osteoporosis and control groups in the combined dataset.

### 2.9. Construction of Regulatory Network

Data sourced from the ChIPBase database [29] made the evaluation of how these transcription factors (TFs) affect their target genes possible. The interactions were then depicted in the mRNA–TF regulatory network, which was mapped using Cytoscape software.

The Comparative Toxicogenomics Database (CTD) [30] was employed to identify both direct and indirect drug targets associated with specific genes, exploring how these genes interact with various drugs.

The relationships of these miRNAs with specific genes were explored using the ENCORI database. Subsequently, the interactions within the mRNA–miRNA regulatory network were depicted through Cytoscape software.

The identification of specific RNA-binding proteins (RBPs) associated with target genes was facilitated using the the StarBase v3.0 database [31] and the resulting mRNA–RBP regulatory network was mapped using Cytoscape software.

The AlphaFold Database [32] (https://www.alphafold.ebi.ac.uk/) contains around 350,000 protein structures predicted by the AlphaFold AI system. Researchers utilized the AlphaFold platform to analyze the protein structures of specific genes and showcase the results.

### 2.10. Statistical Analysis

Analyses were conducted using version 4.1.2 of the R software. Comparisons between groups utilized the Student's *t*-test and the Mann–Whitney *U* test. Additionally, correlation coefficients were calculated through Spearman's method. A *p*-value of less than 0.05 was considered to indicate statistical significance.

### 2.11. Animal Model and Experimental Validation

Osteoporosis Mouse Model: Female C57BL/6 mice (8-weeks old) were randomly divided into control (CON, sham surgery) and osteoporosis (OVX, bilateral ovariectomy) groups (*n* = 5 per group). Surgery was performed under inhaled anesthesia (3% isoflurane induction and 1.5% maintenance). Postoperative care included single housing, standard diet, and controlled environmental conditions (22 ± 2°C, 12-h light/dark cycle). After 2 months, micro-CT confirmed successful osteoporosis induction. Bone tissues were harvested, with left limbs used for histology and right limbs for mRNA extraction. All procedures were approved by the Institutional Animal Ethics Committee (Approval No. IACUC-FPH-SL-20250210 [0638]).

Immunohistochemistry: Paraffin-embedded bone sections were stained with antibodies against C1QBP, CES2, CYB5R4, RAB1B, SERP1, and UFSP2. Staining intensity was quantified using image analysis software under uniform conditions.

RNA Extraction and qRT-PCR: Total RNA was extracted using TRIzol and reverse-transcribed with PrimeScript RT Reagent Kit (TaKaRa). qRT-PCR was performed using SYBR Premix Ex Taq II (Takara) with primers for target genes and 18S rRNA (internal control). Data were analyzed via ΔΔCt method.

## 3. Results

### 3.1. Technology Roadmap

The technology roadmap is detailed in the Supporting Information 2: Figure S1.

### 3.2. Normalization and Batch Effect Correction of Osteoporosis Gene Expression Datasets for Differential Analysis of ERSRGs

The analysis commenced with the normalization of the GSE7429 data using the limma R package. The effectiveness of this preprocessing was assessed by comparing the datasets before and after normalization through box plots (Supporting Information 3: Figure S2A,B). The results from the box plots indicated that the expression levels in GSE7429 were consistently uniform after processing, demonstrating successful normalization and improved comparability of the samples.

Batch effects in the osteoporosis datasets GSE56815 and GSE230665 were addressed using the sva R package. Subsequently, normalization of the aggregated data was conducted with limma. The impact of these adjustments was evaluated through both box plots and PCA (Supporting Information 3: Figure S2C–F). The consistent expression levels across the combined dataset after processing, as indicated by the box plots and PCA results, suggest that the batch effects were effectively mitigated.

### 3.3. Identification of ERSRDEGs in Osteoporosis Using Integrated Gene Expression Datasets

**Figure 1:**
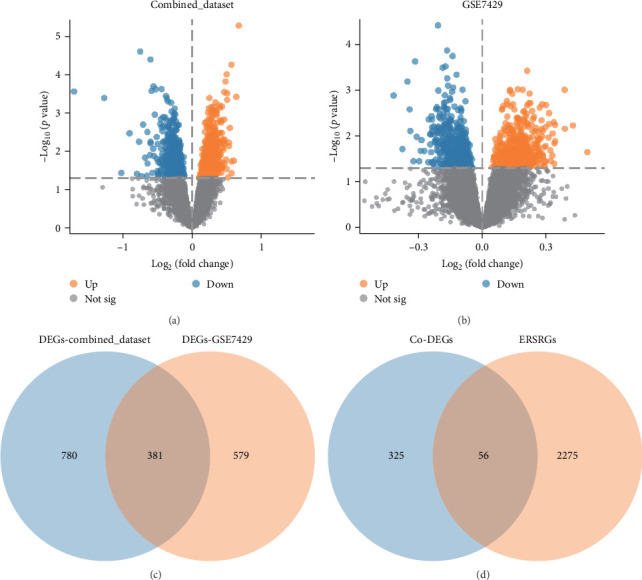
(A) The combined dataset Volcano plot of gene difference analysis between osteoporosis samples and control samples. (B) Volcano plot showing differentially expressed genes between osteoporosis and control samples in the GSE7429 dataset. (C) The combined dataset is the intersection of the differentially expressed genes between osteoporosis samples and control samples and the GSE7429 dataset of differentially expressed genes between osteoporosis samples and control samples. (D) Venn diagram of Co-DEGs and ERSRGs. Co-DEGs, common differentially expressed genes; DEGs, differentially expressed genes; ERSRGs, endoplasmic reticulum stress-related genes.

To compare gene expression between osteoporosis and control groups within the integrated dataset, a differential analysis was performed with limma. This analysis revealed 1161 DEGs that met the threshold of |log fold change (log FC)| > 0 and *p* < 0.05. Of these, 614 genes showed increased expression while 547 exhibited decreased levels. The dataset's analysis outcomes were illustrated in a volcano plot (Figure 1A).

To evaluate gene expression differences in osteoporosis and control samples within the GSE7429 dataset, we employed limma for differential analysis. This identified 960 genes that surpassed the thresholds of |log FC| > 0 and *p* < 0.05. Of these, 461 genes showed increased expression and 499 displayed decreased levels. The outcomes, including a volcano plot (Figure 1B), effectively illustrate the differential analysis results from the GSE7429 dataset.

To pinpoint genes that significantly vary Co-DEGs between osteoporosis and control groups within both the GSE7429 and the merged datasets, 381 Co-DEGs were identified. This was achieved by finding the intersection of DEGs from both the GSE7429 and the combined datasets (Figure 1C).

In order to obtain the ERSRDEGs, the intersection of all Co-DEGs and the ERSRGs was taken and a Venn diagram was drawn (Figure 1D). A total of 56 ERSRDEGs were obtained, namely, MAPK3, STK11, CYB5R4, PIGK, ROR2, CHI3L1, ACSL3, SLC22A13, SERP1, ABT1, PPP2CA, RRAGC, TNFRSF10B, UFSP2, TUBA1B, RPS6KA3, C1QBP, RAB1B, CACYBP, MRPL2, SUCLG2, EHD4, RNF13, CCDC6, G6PC3, ASGR2, CES2, COPS8, SRC, PPP1R13B, H6PD, IFNG, CCT2, GNAI3, NUPR1, GALNT2, CHRNE, EIF2B4, NPHS1, PTP4A1, SCARB1, USP9X, GNE, UBE2I, STAU2, RPL15, TRAF2, GABARAPL1, TNFRSF12A, FDFT1, CYP1A2, NGF, PLD3, STARD5, NR3C1, and MEF2A.

### 3.4. Comprehensive Gene Expression Analysis in Osteoporosis Reveals Enrichment in Key BPs and Pathways

**Figure 2:**
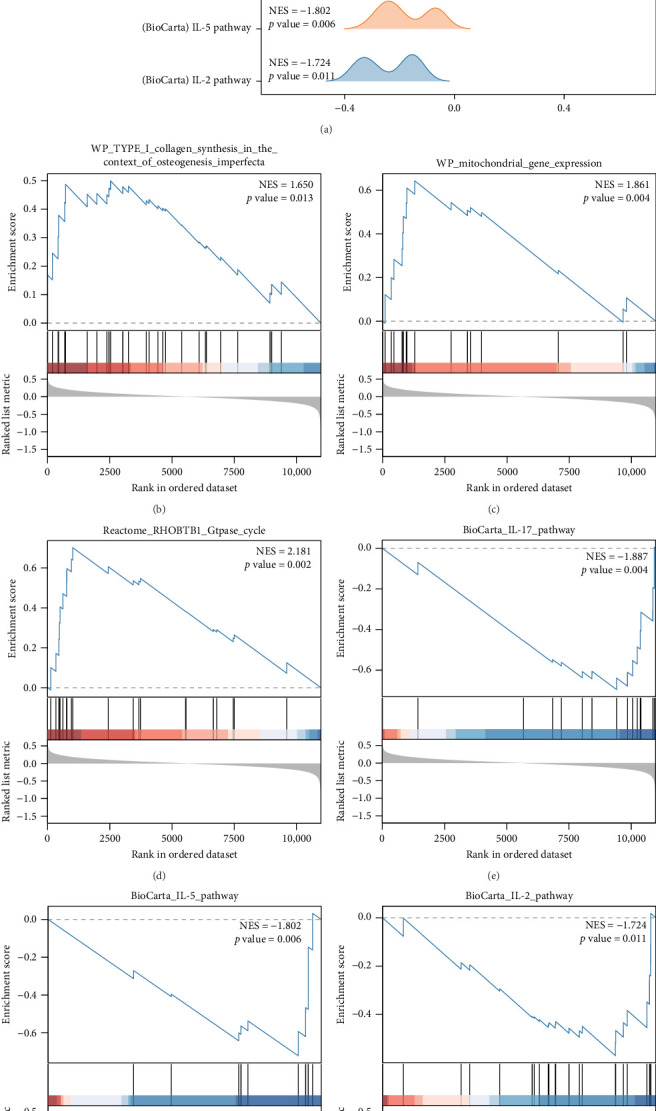
(A) GSEA of the combined dataset shows a mountain plot of six biological functions. (B–G) GSEA shows that ERSRDEGs are significantly enriched in Type I collagen synthesis in the context of osteogenesis imperfecta (B), mitochondrial gene expression (C), RHOBTB1 Gtpase cycle (D), IL-17 pathway (E), IL-5 pathway (F), IL-2 pathway (G). GSEA, gene set enrichment analysis. The screening criterion for GSEA is *p* < 0.05.

All gene expression levels in the combined GEO dataset on osteoporosis, GSEA was used to study the disease control group in the combined GEO dataset. There is a relationship between the expression of all genes and the BP they participate in, the CC they influence, and the MF they exert (Figure 2A). The specific results are shown in Supporting Information 1: Table S3. Genes in the combined dataset were significantly enriched in pathways including Type I collagen synthesis (Figure 2B), mitochondrial gene expression (Figure 2C), RHOBTB1 Gtpase cycle (Figure 2D), Il-17 pathway (Figure 2E), IL-5 pathway (Figure 2F), IL-2 pathway (Figure 2G), and other biologically relevant functions and signaling pathways.

### 3.5. GSVA Identifies Significantly Enriched Pathways in Osteoporosis

**Figure 3:**
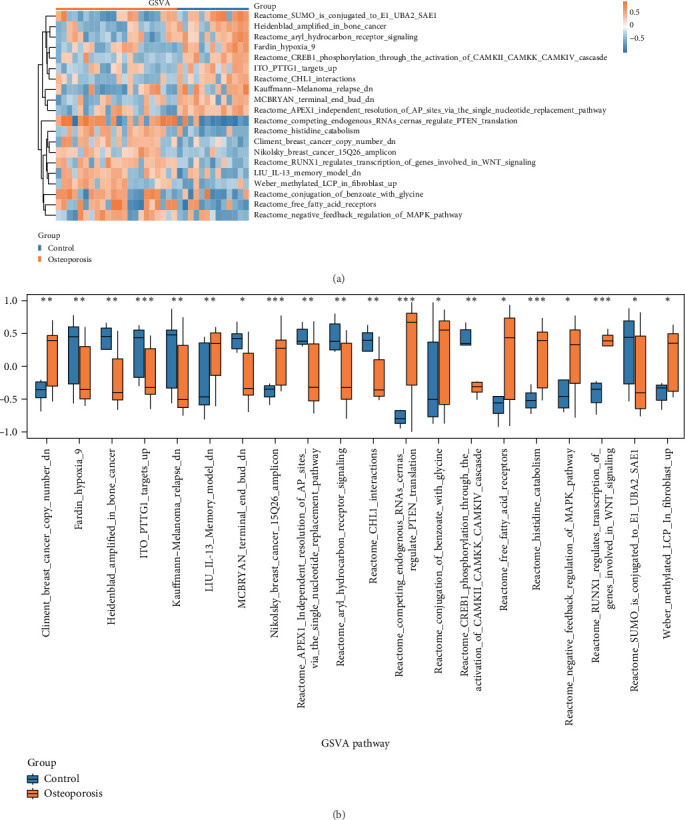
Group comparison plot (A) and heat map (B) of GSVA results between osteoporosis samples and control samples in the osteoporosis dataset. GSVA, gene set variation analysis. In the group comparison chart, the symbol ns is equivalent to *p* ≥ 0.05, no statistical significance; *⁣*^*∗*^ represents *p* < 0.05, statistically significant; *⁣*^*∗∗*^ represents *p* < 0.01, highly statistically significant; *⁣*^*∗∗∗*^ represents *p* < 0.001, highly statistically significant. The screening criteria for GSVA were |log FC| > 0.0 and *p* < 0.05. Blue represents the combined dataset control sample and orange represents the combined dataset osteoporosis sample.

GSVA analysis was conducted on all the genes in the combined dataset comparing disease control groups. Specific information can be found in Supporting Information 1: Table S4. Following this, pathways that showed statistical significance (*p* < 0.05) and had top 10 positively enriched and top 10 negatively enriched rankings of log FC were identified and visualized in group comparison diagrams (Figure 3A). The GSVA results indicated enrichment in various pathways. These findings were statistically significant (*p* < 0.05) in samples from individuals with osteoporosis and control samples. Last, based on the GSVA results, the expression differences in 20 pathways between the osteoporosis samples and the control sample groups were analyzed and presented visually using heat maps (Figure 3B).

### 3.6. Comprehensive Enrichment and Network Analysis of ERSRDEGs Unravel Key Pathways in Osteoporosis

**Figure 4:**
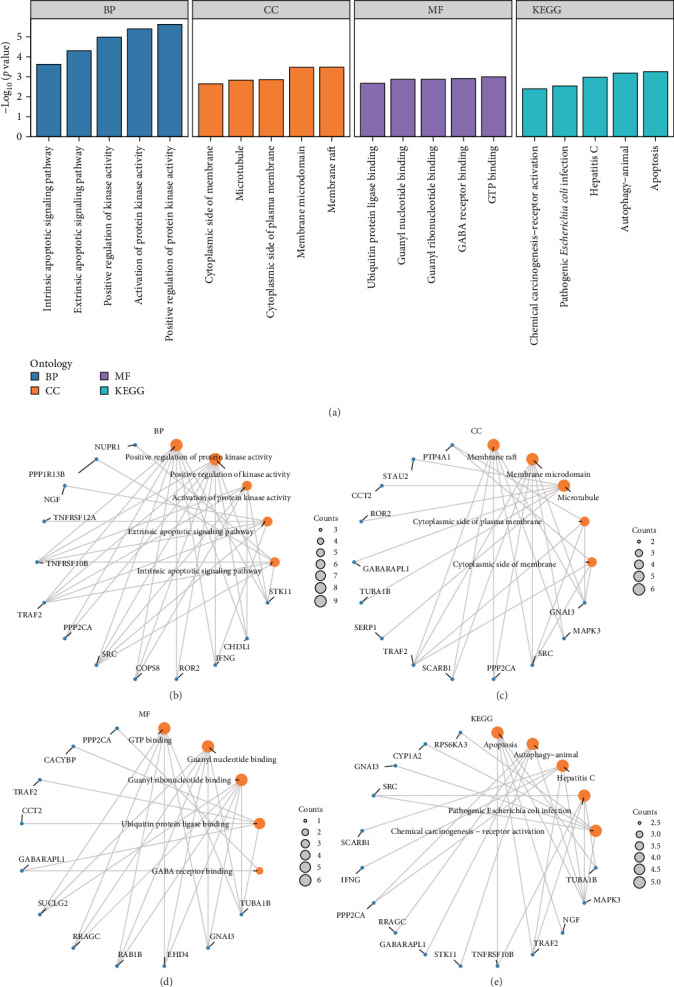
(A) GO and KEGG enrichment analysis results of ERSRDEGs: BP, CC, MF, and biological pathways. The abscissa is GO terms and KEGG terms. (B–E) Network diagram showing the results of GO and KEGG enrichment analysis of ERSRDEGs: BP (B), CC (C), MF (D), and KEGG (E). Orange nodes represent entries, green nodes represent molecules, and connecting lines represent the relationship between entries and molecules. BP, biological process; CC, cellular component; ERSRDEGs, endoplasmic reticulum stress-related differentially expressed genes; GO, Gene Ontology; KEGG, Kyoto Encyclopedia of Genes and Genomes; MF, molecular function. The screening criteria for GO and pathway KEGG enrichment analysis were *p* < 0.05.

Through GO and KEGG enrichment analysis, the BP, CC, and MF of 56 ERSRDEGs were further explored and the relationship between biological pathways and osteoporosis. The specific results are shown in Supporting Information 1: Table S5. The results show that 56 ERSRDEGs are mainly enriched in positive regulation of protein kinase activity, positive of kinase activity, activation of protein kinase activity, and extrinsic apoptotic signaling pathway, intrinsic apoptotic signaling pathway and other BP; membrane raft, membrane microdomain, microtubule, cytoplasmic side of plasma membrane, cytoplasmic side of membrane and other CC; GTP binding, guanyl nucleotide binding, guanyl ribonucleotide binding, ubiquitin protein ligase binding, GABA receptor binding, and other MF. At the same time, it is also enriched in apoptosis, autophagy-animal, hepatitis C, pathogenic *Escherichia coli* infection, chemical carcinogenesis-receptor activation, and other KEGG. The results of GO and KEGG enrichment analysis were visualized through histograms (Figure 4A).

A circular diagram representing the network of BP, CC, MF, and biological pathways was created based on the results of GO and KEGG enrichment analysis (Figure 4). The connecting line displays the associated molecule along with the annotation of its respective entry. The size of each node within the diagram corresponds to the number of molecules contained in the entry.

Finally, pathway KEGG enrichment analysis of Chemical carcinogenesis-receptor activation (Supporting Information 4: Figure S3A), pathogenic *Escherichia coli* infection (Supporting Information 4: Figure S3B), hepatitis C (Supporting Information 4: Figure S3C), autophagy-animal (Supporting Information 4: Figure S3D), and apoptosis (Supporting Information 4: Figure S3E) was performedvia the R package Pathview Perform a visual display of the pathway diagram.

### 3.7. Development and Validation of a Diagnostic Model for Osteoporosis Based on ERSRDEGs

**Figure 5:**
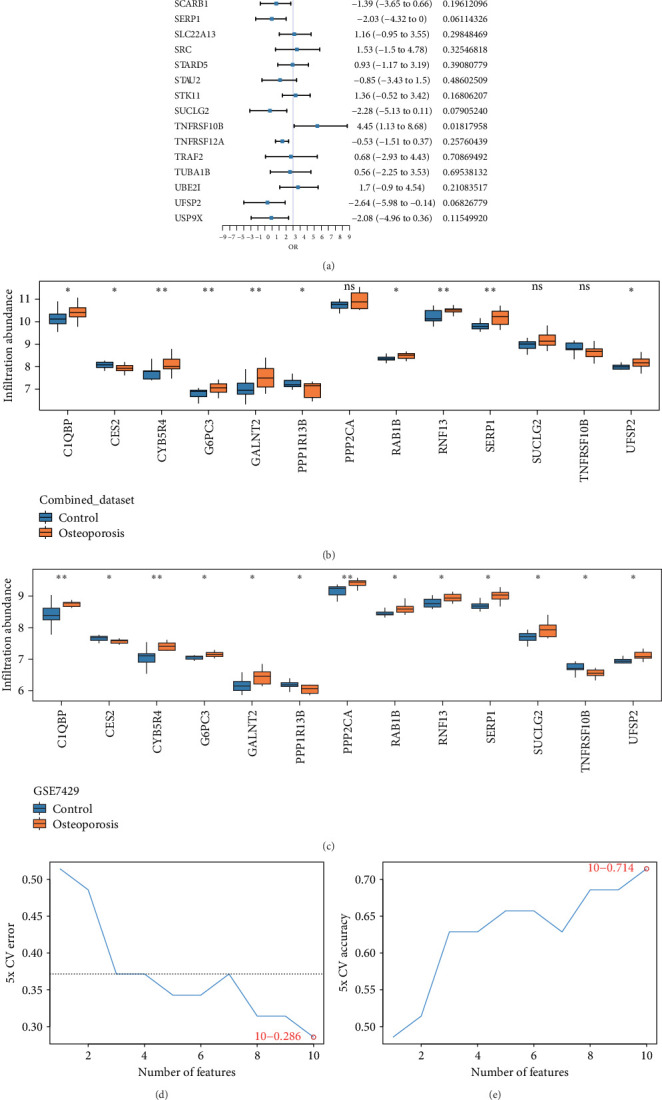
(A) Forest plot of logistic regression model results. (B, C) Group comparison diagram of ERSRDEGs between the osteoporosis group and the normal group in the combined GEO dataset (B) and the GSE7429 dataset (C). (D, E) Visualization of the number of genes with the lowest error rate (D) and the number of genes with the highest accuracy (E) obtained by the SVM algorithm. (F, G) Diagnostic model plot (F) and variable trajectory plot (G) of the LASSO regression model. (H) Venn diagram of model ERSRDEGs included in the LASSO regression model and in the SVM. (I) Boxplot diagram display of functional similarity analysis of model genes. In the group comparison chart, the symbol ns is equivalent to *p* ≥ 0.05, which has no statistical significance; the symbol “*⁣*^*∗*^” represents *p* < 0.05, which is statistically significant; the symbol “*⁣*^*∗∗*^” is equivalent to *p* < 0.01, which is highly statistically significant; the symbol “*⁣*^*∗∗∗*^” equivalent to *p* < 0.001, highly statistically significant. ERSRDEGs, endoplasmic reticulum stress-related differentially expressed genes; LASSO, least absolute shrinkage and selection operator; SVM, support vector machine.

In order to determine the diagnostic value of 56 ERSRDEGs in osteoporosis, a logical regression was conducted, constructing a logistic regression model and displaying it visually through forest plot (Figure 5A). The results showed that 13 ERSRDEGs were statistically significant (*p* < 0.1) in the logistic regression model, namely, C1QBP, CES2, CYB5R4, G6PC3, GALNT2, PPP1R13B, PPP2CA, RAB1B, RNF13, SERP1, SUCLG2, TNFRSF10B, and UFSP2.

Thirteen distinct ERSRDEGs were identified and comparative group diagrams were generated for visualization purposes (Figure 5B,C). Utilizing the group comparison data, only those ERSRDEGs displaying noteworthy variances between the osteoporosis and normal groups across both sets of data were included. Ultimately, 10 ERSRDEGs met this criterion: CYB5R4, RAB1B, UFSP2, GALNT2, RNF13, SERP1, CES2, G6PC3, PPP1R13B, and C1QBP.

A SVM model was created using 10 ERSRDEGs and the SVM algorithm. The model determined the genes with the lowest error rate (Figure 5D) and the highest accuracy rate (Figure 5E). The findings indicate that with 10 genes, the SVM model achieves peak accuracy.

A LASSO regression model was constructed based on 10 ERSRDEGs through LASSO regression analysis. The LASSO regression model diagram (Figure 5F) and the LASSO variable trajectory diagram (Figure 5G) were plotted for visual representation. The results showed that the LASSO regression model included seven ERSRDEGs: CYB5R4, RAB1B, UFSP2, RNF13, SERP1, CES2, and C1QBP.

The genes selected for the LASSO regression model overlapped with those chosen for the SVM, resulting in the identification of model genes through the creation of a Venn diagram. The coefficients of the model genes in the LASSO regression model were then combined with the expression data from the combined dataset to obtain the osteoporosis diagnostic model and the corresponding risk score. The risk score was calculated as follows:

  $$\begin{array}{c} \text{Risk score}=-92.92863372+\text{CYB5R4}\times 2.289953309+\text{SERP1}\times 1.035150235+\text{C1QBP}\times 0.296414099+\text{CES2}\times -1.177135117+\text{RAB1B}\times 4.94123259+\text{RNF13}\times 1.499424138+\text{UFSP2}\times 1.77301333. \end{array}$$

In conclusion, we utilized the R software package GOSemSim to assess the semantic similarities among the GO terms, collections of GO terms, gene products, and gene sets of the seven model genes. Subsequently, a boxplot was generated to visualize the results. The results indicated that SERP1 exhibited the highest functional similarity score among the model genes.

### 3.8. Validation and Clinical Utility Assessment of ERSRGs Signature for Osteoporosis Diagnosis

To validate the value of the osteoporosis diagnostic model, a nomogram was created using the model genes to illustrate their relative importance in the combined dataset (Supporting Information 5: Figure S4A). The results indicate that the expression level of the CYB5R4 gene has a notably higher influence on the osteoporosis diagnostic model compared to other factors, while the expression level of the CES2 gene has a lower impact on the osteoporosis diagnostic model relative to other factors.

The clinical utility of the osteoporosis diagnostic model was assessed using DCA on the combined dataset (Supporting Information 5: Figure S4B). The results indicate that the model consistently outperforms both positive and negative controls within a specific range, resulting in higher net benefit and improved clinical utility. Furthermore, the ROC curve generated using the R package pROC for the combined dataset (Supporting Information 5: Figure S4C) demonstrates high accuracy of the risk score (AUC > 0.9) across various groups.

Based on the expression levels of the seven model genes in the GSE7429 dataset, a calculation formula was applied to determine the risk score of the osteoporosis diagnostic model. The risk score was then used to evaluate the accuracy of the model in predicting osteoporosis within the GSE7429 dataset. By utilizing the R package pROC, a ROC curve was generated based on the risk score, demonstrating high accuracy (AUC > 0.9) in distinguishing between different groups within the GSE7429 dataset (Supporting Information 5: Figure S4D). This indicates that the osteoporosis diagnostic model is effective in accurately identifying individuals at risk for osteoporosis within this specific dataset.

### 3.9. GSEA Reveals Distinct BP and Signaling Pathways Associated With High and Low Risk of Osteoporosis Based on ERSRGs Signature

Initially, only samples with osteoporosis were selected, and the median risk score expression from the osteoporosis diagnostic model based on the combined dataset was used to divide the osteoporosis samples into two groups: high-risk (high) and low-risk (low).

We performed a differential gene expression analysis on all genes in the combined dataset using the limma package. The comparison was carried out between the high-risk (high) and low-risk (low) groups. The results obtained from this analysis were then used in GSEA to investigate the association between gene expression in the high and low-risk groups and their significance in BP, CC, and MF. By examining these associations, we can better understand the mechanisms underlying the observed differences between the high and low-risk groups. The specific results are presented in Supporting Information 1: Table S6. The results show that genes within the combined dataset are significantly enriched in biologically relevant functions and signaling pathways, including the IL-2–PI3K pathway (Supporting Information 6: Figure S5C), photodynamic therapy-induced unfolded protein response (UPR; Supporting Information 6: Figure S5D), IL-18 signaling pathway (Supporting Information 6: Figure S5E), UPR (Supporting Information 6: Figure S5F), intra-golgi and retrograde golgi-to-ER traffic (Supporting Information 6: Figure S5G), golgi-to-ER retrograde transport (Supporting Information 6: Figure S5H), and others.

### 3.10. GSVA Identifies Significantly Enriched Pathways Associated With High and Low Risk of Osteoporosis Based on ERSRGs Signature

The combined dataset was analyzed using GSVA to compare gene expression levels between high and low risk groups. See Supporting Information 1: Table S7 for specific information. Subsequently, the top 10 positively and negatively correlated enriched pathways were identified using a significance level of *p* < 0.05 and log FC ranking. The findings were then visualized using a comparative chart (Supporting Information 7: Figure S6A). The GSVA results revealed significant enrichment (*p* < 0.05) of the following pathways in both high-risk and low-risk sample groups: Samols targets of KHSV miRNAs up, Tomida lung cancer poor survival, Wang esophagus cancer progression up, Nikolsky breast cancer 1q32 amplicon, Yamanaka glioblastoma survival up, Bierie inflammatory response TGFB1, attachment of GPI anchor to UPAR, CREB3 factors activate genes, Jones TCOF1 targets, Turjanski MAPK11 targets, Serotonin and melatonin biosynthesis, Tesar ALK and JAK targets mouse ES d4 dn, Fructose catabolism, Biocarta PELP1 pathway, Schlesinger methylated in colon cancer, Eicosanoid metabolism via cytochrome P450 monooxygenases pathway, Lopez mesothelioma survival worst vs. best dn, Tonks targets of RUNX1 RUNX1T1 fusion sustained in granulocyte dn, amino acid conjugation, and conjugation of benzoate with glycine. Following the GSVA analysis, a differential expression analysis of the 20 pathways was conducted between osteoporosis and control samples. The results were then visualized using heat maps (Supporting Information 7: Figure S6B).

### 3.11. Immune Cell Infiltration Patterns and Correlation With ERSRGs Across High- and Low-Risk Osteoporosis Samples

Initially, only osteoporosis samples were selected, and the median risk score expression from the osteoporosis diagnostic model in the combined dataset was used to classify the osteoporosis samples into high-risk (high) and low-risk (low) categories.

**Figure 6:**
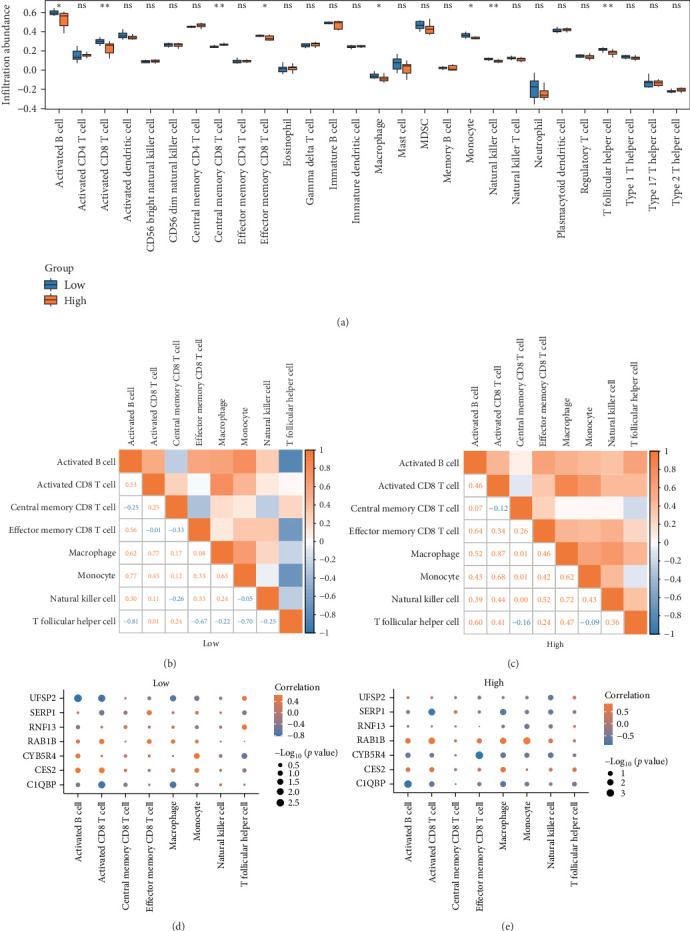
(A) Group comparison chart of immune cells in high-risk and low-risk group samples in the combined dataset. (B, C) Correlation heat map of immune cell infiltration abundance in high-risk (B) and low-risk (C) group samples in the combined GEO dataset. (D, E) Bubble chart of the correlation between model genes and immune cell infiltration abundance in high-risk and low-risk group samples in the combined GEO dataset. ssGSEA, single-sample gene set enrichment analysis. In the group comparison chart, “ns” represents *p* ≥ 0.05, indicating no statistical significance; *⁣*^*∗*^ represents *p* < 0.05, indicating statistical significance; *⁣*^*∗∗*^ represents *p* < 0.01, indicating high statistical significance; and *⁣*^*∗∗∗*^ represents *p* < 0.001, indicating extreme statistical significance. The absolute value of the correlation coefficient (*r*) indicates the strength of the correlation: below 0.3 suggests a weak or irrelevant correlation, between 0.3 and 0.5 suggests a weak correlation, between 0.5 and 0.8 suggests a moderate correlation, and above 0.8 suggests a strong correlation.

The abundance of immune cell infiltration for 28 different types of immune cells was calculated using the combined GEO dataset and the ssGSEA algorithm. First, the expression matrix was used to identify variations in the abundance of immune cell infiltration across different groups. The group comparison charts show significant differences in the infiltration levels of eight types of immune cells in the bone, including activated B cells, activated CD8^+^ T cells, central memory CD8^+^ T cells, effector memory CD8^+^ T cells, macrophages, monocytes, NK cells, and T follicular helper cells. The group comparison chart (Figure 6A) demonstrates a statistically significant difference between the control and experimental groups (*p* < 0.05).

The infiltration abundance of eight immune cell types in samples from both high-risk and low-risk groups was analyzed using correlation heat maps (Figure 6B,C). The results revealed predominantly positive correlations among the immune cells. These findings suggest that changes in the abundance of one immune cell type are associated with changes in the abundance of other immune cell types. This information may be valuable for understanding the interactions and dynamics of immune cell populations in different risk groups.

Subsequently, an analysis was performed to investigate the relationship between seven model genes and eight immune cells in both the high-risk and low-risk group samples within the combined dataset. The findings were then visualized using a correlation bubble chart (Figure 6D,E). The results show that among the samples from the low-risk group, the ERSRDEG UFSP2 exhibited the strongest positive correlation with activated B cells (*r* = −0.818181818). Among the samples from the high-risk group, the strongest correlation was observed between the ERSRDEG CYB5R4 and effector memory CD8^+^ T cells (*r* = −0.863636364).

**Figure 7:**
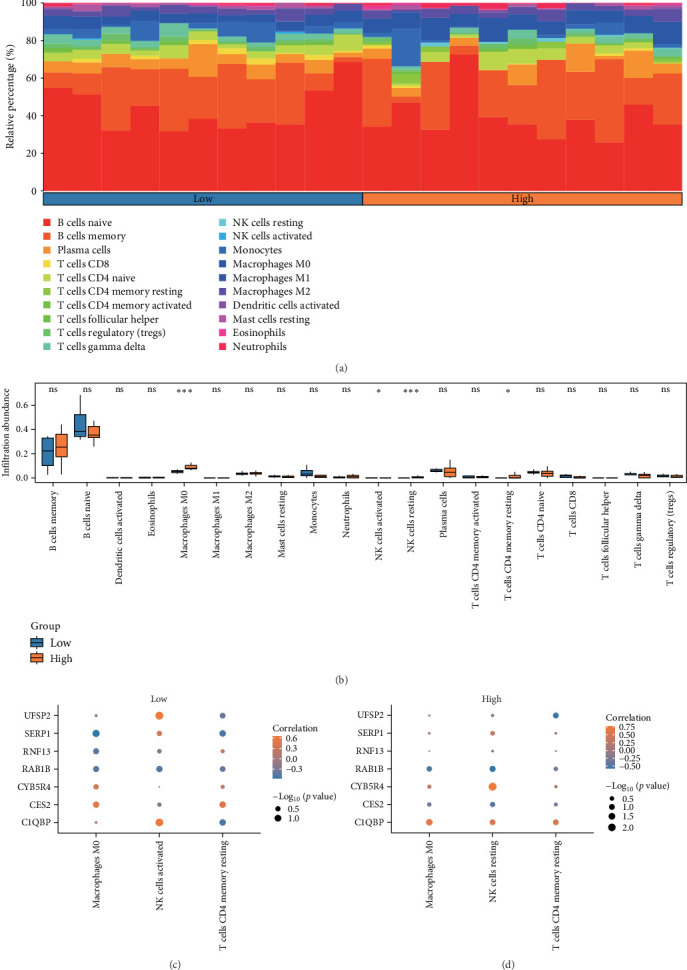
(A) Histogram and (B) group comparison chart of the proportion of immune cells in high-risk and low-risk group samples in the combined dataset. (C, D) Bubble charts of the correlation between model genes and immune cell infiltration abundance in high-risk and low-risk group samples in the combined GEO dataset. In the group comparison chart, “ns” represents *p* ≥ 0.05, indicating no statistical significance; *⁣*^*∗*^ represents *p* < 0.05, indicating statistical significance; *⁣*^*∗∗*^ represents *p* < 0.01, indicating high statistical significance; and *⁣*^*∗∗∗*^ represents *p* < 0.001, indicating extreme statistical significance. The absolute value of the correlation coefficient (*r*) between 0.3 and 0.5 suggests a weak correlation, while between 0.5 and 0.8 suggests a moderate correlation.

The abundance of 22 distinct immune cell types was estimated using the CIBERSORT algorithm on the combined dataset. After analyzing the immune cell infiltration, a bar graph was generated to visualize the distribution of immune cells in the combined dataset (Figure 7A). Subsequently, a diagram comparing the differences in immune cell infiltration abundance between high-risk and low-risk groups in the combined dataset was presented (Figure 7B). The results revealed significant differences in the abundance of three distinct immune cell types between high-risk and low-risk group samples in the combined GEO dataset (*p* < 0.05). The immune cell types with significant differences were M0 macrophages, activated NK cells, and resting memory CD4^+^ T cells.

Bubble charts were used to visualize the correlation between the abundance of immune cell infiltration and model genes in the high-risk and low-risk groups, utilizing data from the combined GEO dataset (Figure 7C,D). The findings revealed that among the samples from the low-risk group, the ERSRDEG UFSP2 showed the strongest positive correlation with activated NK cells (*r* = 0.642283206). In the high-risk group samples, the ERSRDEG CYB5R4 exhibited the strongest correlation with resting NK cells (*r* = 0.770674636).

### 3.12. Integrative Network Analysis of Transcriptional and Posttranscriptional Regulation in Model Genes

**Figure 8:**
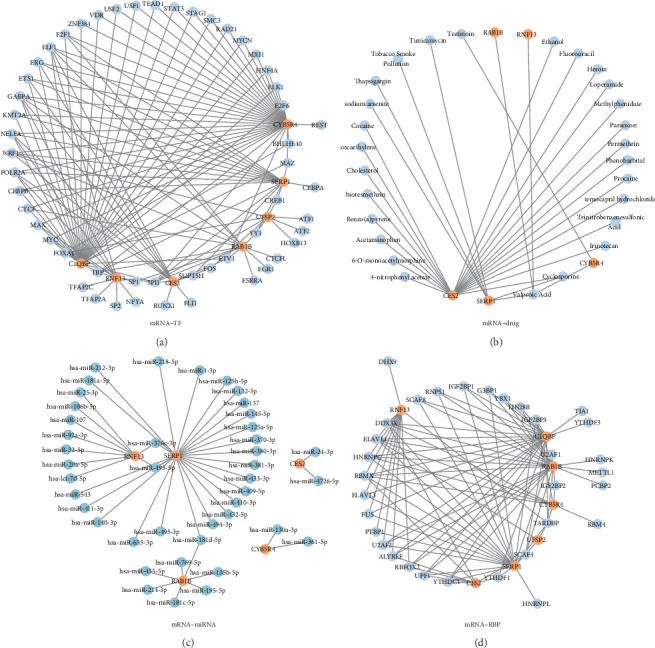
(A) mRNA–TF regulatory network of model genes. (B) mRNA–drug regulatory network of model genes. (C) mRNA–miRNA regulatory network of model genes. (D) mRNA–RBP regulatory network of model genes. RBP, RNA-binding protein; TF, transcription factor. In the mRNA–TF regulatory network, orange nodes represent mRNAs and blue nodes represent TFs. In the mRNA–drug regulatory network, orange nodes represent mRNAs and blue nodes represent drugs. In the mRNA–miRNA regulatory network, orange nodes represent mRNAs and blue nodes represent miRNAs. In the mRNA–RBP regulatory network, orange nodes represent mRNAs and blue nodes represent RBPs.

TFs influence gene regulation at the post-transcriptional level by binding to specific genes. Model genes and their associated TFs were obtained from the ChIPBase database. Using Cytoscape software (Figure 8A), a visual representation of the mRNA–TF regulatory network was constructed. The resulting network consists of 52 TFs and seven model genes (Supporting Information 1: Table S8).

The miRNAs significantly impact the regulation of gene expression, playing a key role across biological development and evolutionary processes. It is common for several miRNAs to control a single gene. The miRNAs associated with the model genes were obtained from the TarBase database, and an mRNA–miRNA interaction network was constructed and visualized using the Cytoscape software (Figure 8C). The network consists of five model genes and 42 miRNAs. Detailed information is provided in Supporting Information 1: Table S10.

The CTD was used to identify potential drugs or molecular compounds associated with the model genes. The mRNA–drug regulatory network was first constructed and then visualized using Cytoscape software (Figure 8B). The network includes five model genes and 27 drugs or molecular compounds. Detailed information can be found in Supporting Information 1: Table S9.

RBPs [33] were pivotal in gene regulation by influencing essential BPs such as RNA synthesis, editing, modification, transport, and decoding. The StarBase database was used to predict RBPs associated with the model genes. The mRNA–RBP regulatory network was then constructed and visualized using the Cytoscape software (Figure 8D). The network consists of seven model genes and 32 RBPs. Detailed information is provided in Supporting Information 1: Table S11.

Finally, the protein structures of the seven model genes were analyzed using the AlphaFold website, and the results were displayed (Supporting Information 8: Figure S7A–G).

### 3.13. Integrative Network Analysis of Transcriptional and Posttranscriptional Regulation in Model Genes

**Figure 9:**
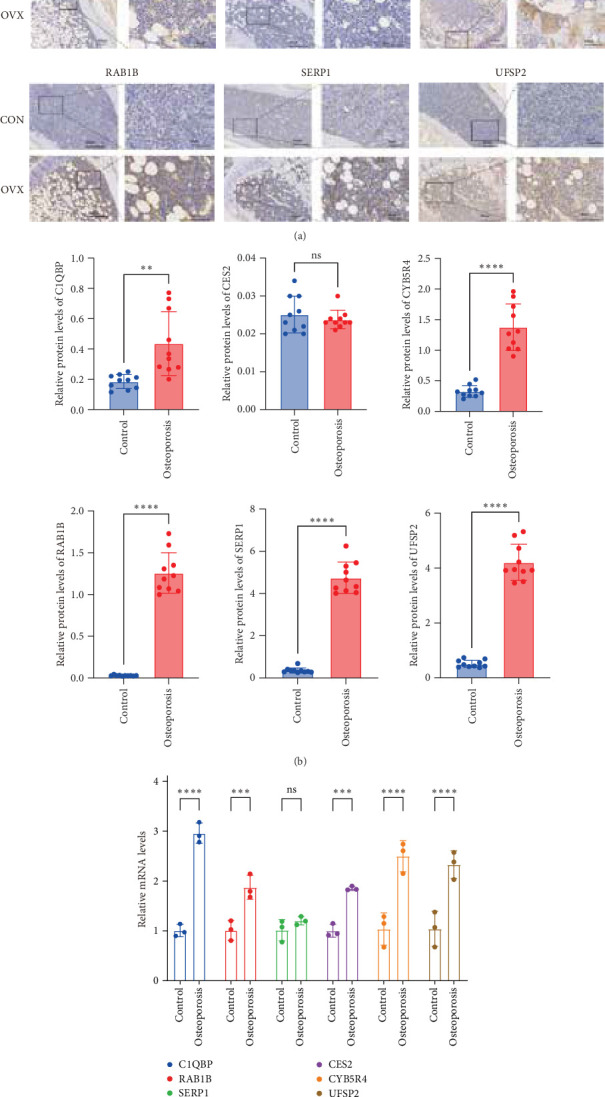
(A) Immunohistochemical staining of C1QBP, CES2, CYB5R4, RAB1B, SERP1, and UFSP2 in CON and OVX groups (scale bars: 100 μm low-magnification and 50 μm high-magnification). (B) Semiquantitative analysis of protein expression. (C) mRNA levels measured by qRT-PCR. ns, not significant; ⁣^*∗∗*^*p* < 0.01; ⁣^*∗∗∗∗*^*p* < 0.0001.

Immunohistochemistry revealed significant upregulation of C1QBP, CYB5R4, RAB1B, SERP1, and UFSP2 proteins in OVX mice compared to controls(*p* < 0.01), while CES2 showed no significant change (Figure 9A,B). qRT-PCR demonstrated concordant mRNA upregulation for C1QBP, CYB5R4, RAB1B, and UFSP2 (*p* < 0.01). Notably, CES2 mRNA was elevated in OVX mice (*p* < 0.001), contrasting with unchanged protein levels, whereas SERP1 mRNA remained unaltered despite increased protein expression (Figure 9C).

## 4. Discussion

Osteoporosis is a severe condition characterized by reduced bone density and deterioration of bone microarchitecture, resulting in increased fracture risk and frailty [34]. Osteoporosis poses a significant threat to public health, affecting millions of individuals worldwide, particularly postmenopausal women and the elderly population [35]. The asymptomatic nature of osteoporosis frequently results in delayed diagnosis, often when patients present with painful fractures that can severely diminish their quality of life and lead to high healthcare costs [36]. Although treatments are available to reduce bone loss and prevent fractures, their efficacy is limited, emphasizing the urgent need for novel therapeutic approaches and a better understanding of the underlying disease mechanisms [37].

ERS plays a critical role in numerous diseases by influencing cell function and survival through mechanisms such as the UPR and autophagy. Studies in other diseases have emphasized the therapeutic potential of targeting ERS pathways [38, 39]. Recent investigations have begun to reveal the complex interplay between ERS and bone metabolism, indicating that ERS may contribute to osteoporosis by altering bone remodeling processes and promoting apoptosis in osteoblasts and osteocytes [40]. However, translating these findings to osteoporosis is still in its early stages. This study aims to investigate the correlation between ERSRGs and osteoporosis, with the objective of elucidating the impact of ERS on disease progression.

This study analyzed gene expression data from individuals diagnosed with osteoporosis and compared it to a healthy control group. The analysis identified DEGs. The potential of these DEGs as diagnostic markers was evaluated using ROC curve analysis, which identified specific genes with high diagnostic value in differentiating patients from controls. Subsequent GO and KEGG enrichment analyses revealed that the DEGs were primarily associated with essential BPs such as apoptosis and the regulation of enzymatic activity in proteins. Moreover, GSEA highlighted the significance of pathways related to inflammatory response and cell cycle regulation in the pathogenesis of osteoporosis. LASSO regression was employed to identify the most prognostically relevant genes among the DEGs. A Cox regression model, incorporating selected genes, was constructed and visualized as a nomogram. This model serves as a valuable tool for clinicians to improve the accuracy of patient prognostic predictions. DCA further validated the model's accuracy and resolution. These results offer valuable insights for discovering new therapeutic targets. Consensus clustering analysis identified molecular subtypes of osteoporosis based on DEGs, revealing differences in immune cell infiltration among the subtypes and offering novel insights into disease heterogeneity and personalized treatment approaches. These findings not only advance our understanding of the molecular mechanisms underlying osteoporosis but also lay the foundation for future clinical applications and therapeutic strategies.

Moreover, our analysis revealed enrichment in inflammation-related pathways, such as those associated with hepatitis C and pathogenic *Escherichia coli* infection, highlighting their potential relevance to osteoporosis [41, 42]. GSEA confirmed significant enrichment in pathways crucial for osteoporosis development, including mitochondrial gene expression, RHOBTB1 GTPase cycle, and the IL-17 pathway. These findings emphasize the importance of cellular energy metabolism, cytoskeletal regulation, and immune-inflammatory responses in osteoporosis pathogenesis. Mitochondrial gene expression, which is critical for cellular energy metabolism, can influence osteoblast and osteoclast activity when dysregulated, consequently affecting bone remodeling processes [43]. The detection of mitochondrial dysfunction in our dataset implies its potential role in the etiology of osteoporosis, presenting possible therapeutic targets. The RHOBTB1 GTPase cycle, which is essential for cytoskeletal organization and cellular trafficking, facilitates osteoblast function and bone formation [44, 45]. The enrichment of the RHOBTB1 GTPase cycle suggests its potential involvement in mechanisms regulating bone density and strength, which are frequently impaired in osteoporosis.

The immune cell infiltration analysis in our study revealed significant differences in immune cell abundance across various risk groups for osteoporosis. This highlights the importance of understanding the complex interplay between the immune system and bone health for enhanced prevention and management of osteoporosis. ssGSEA showed increased levels of activated B cells and CD8^+^ T cells in high-risk groups, resembling patterns observed in tumor microenvironments. Interestingly, a strong negative correlation was found between UFSP2 and activated B cells (*r* = −0.82) and between CYB5R4 and effector memory CD8^+^ T cells (*r* = −0.86) in low-risk groups, implying that these cells may influence osteoporosis development through gene regulation. The CIBERSORT analysis confirmed differential infiltration of M0 macrophages, activated NK cells, and resting CD4^+^ memory T cells, consistent with Newman et al.'s [28] findings on immune cell infiltration in various cancer types using the CIBERSORT algorithm.

Our team developed a diagnostic model for osteoporosis by leveraging ERSRDEGs and performing logistic regression analysis. Our approach is consistent with Varadi et al.'s [32] work on employing the AlphaFold database for protein structure prediction. By employing the SVM algorithm and LASSO regression analysis, we identified crucial genes for osteoporosis diagnosis. Our methodology not only takes into account gene expression levels but also improves model generalization by utilizing LASSO regression to mitigate overfitting. Furthermore, we utilized nomograms and DCA as powerful tools for assessing clinical prediction models, as shown in Stelzer et al.'s [12] study. ROC curve analysis revealed high diagnostic accuracy (AUC > 0.9), in agreement with Xie et al.'s [9] results on osteoporosis diagnostic markers. These findings provide novel biomarkers for early identification of disease risk and lay the foundation for developing diagnostic models for related diseases in the future.

Our study investigated the complex roles of key genes in osteoporosis, elucidating their functions and regulatory mechanisms. The construction of diverse networks, such as protein–protein interaction (PPI), ceRNA, mRNA–miRNA, mRNA–TF, and mRNA–drug interactions, yielded significant findings. Varadi et al. [32] demonstrated CYB5R4′s involvement in cholesterol regulation in cardiovascular diseases. Our findings suggest that CYB5R4 may contribute to osteoporosis pathogenesis by modulating lipid metabolism and oxidative stress in bone cells. Prior research has highlighted MAPK3's critical role in cell proliferation, differentiation, and stress response. CYP1A2, a crucial enzyme in drug metabolism, influences drug bioavailability and toxicity [46]. Our study revealed significant interactions between CYP1A2 and various drugs, suggesting its potential impact on the efficacy of osteoporosis treatments. CES2, a liver-specific enzyme, plays a vital role in the metabolism of drugs and endogenous compounds [47]. Alterations in CES2 expression in osteoporosis may indicate changes in bone marrow adiposity, influencing bone turnover and providing insights into the metabolic interplay within bone tissue.

Our bioinformatics findings were experimentally validated in an ovariectomy-induced osteoporosis model. The upregulation of C1QBP, CYB5R4, RAB1B, SERP1, and UFSP2 at both transcriptional and translational levels underscores their roles in ERS-mediated bone loss. The discordance between CES2 mRNA and protein levels suggests posttranscriptional regulation, such as miRNA-mediated repression or protein degradation, warranting further investigation. Similarly, the lack of SERP1 mRNA changes despite elevated protein hints at translational efficiency modulation. These results reinforce the diagnostic potential of the identified ERSRDEGs and highlight the complexity of gene expression regulation in osteoporosis.

The integration of bioinformatics and experimental approaches bridges computational predictions with biological relevance. Future studies should explore mechanistic roles of these genes in ERS pathways and validate the diagnostic model in clinical cohorts.

When considering the limitations of this study, the analysis in this study was limited by the use of a small sample size, which may restrict the generalizability of these findings to a larger population. The diagnostic models and risk score systems developed in this study were not clinically validated, a step that is essential for translating these findings into clinical practice. Moreover, the utilization of multiple datasets might introduce batch effects, although efforts were made to mitigate these effects through data preprocessing steps.

## 5. Conclusion

This study identified ERS-related ERSRDEGs as key contributors to osteoporosis, validated through bioinformatics and experimental approaches. Diagnostic models utilizing logistic regression and SVM, optimized by LASSO, achieved high accuracy (AUC > 0.9), while immune infiltration analysis revealed distinct immune cell profiles linked to disease risk. Experimental validation in an ovariectomy-induced mouse model confirmed significant upregulation of C1QBP, CYB5R4, RAB1B, SERP1, and UFSP2 at both mRNA and protein levels, reinforcing their biomarker potential. Notably, CES2 exhibited discordant mRNA-protein expression, suggesting posttranscriptional regulation. These findings, supported by mapped regulatory networks involving TFs and miRNAs, highlight the translational potential of ERSRDEGs for precision diagnostics and therapies, underscoring the need for integrating computational and experimental strategies in osteoporosis research.

## Data Availability

The data that support the findings of this study are available from the corresponding author upon reasonable request.

## Nomenclature

ERSRDEGs: Endoplasmic reticulum stress-related genes
GEO: Gene Expression Omnibus
GSEA: Gene set enrichment analysis
GSVA: Gene set variation analysis
GO: Gene Ontology
KEGG: Kyoto Encyclopedia of Genes and Genomes
SVM: Support vector machine
LASSO: Least absolute shrinkage and selection operator
DEGs: Differentially expressed genes
DXA: Dual-energy X-ray absorptiometry
BMD: Bone mineral density
CTX: C-terminal telopeptide of Type I collagen
ELISA: Enzyme-linked immunosorbent assay
qPCR: Real-time quantitative polymerase chain reaction
qRT-PCR: Quantitative reverse transcription polymerase chain reaction
ER: Endoplasmic reticulum
PCA: Principal component analysis
Co-DEGs: Common differentially expressed genes
MSigDB: Molecular Signatures Database
BP: Biological process
CC: Cell component
MF: Molecular function
DCA: Decision curve analysis
ROC: Receiver operating characteristic
AUC: Area under the ROC curve
SsGSEA: Single-sample gene-set enrichment analysis
TF: Transcription factors
CTD: Comparative Toxicogenomics Database
RBP: RNA-binding protein
UPR: Unfolded protein response.

## Supporting Information

Supporting Information 1 Table S1: Osteoporosis datasets information list. Table S2: List of ERSRGs. Table S3: GSEA enrichment analysis results of combined dataset control-osteoporosis group genes. Table S4: GSVA enrichment analysis results of combined dataset control-osteoporosis group genes. Table S5: GO and KEGG enrichment analysis results. Table S6: GSEA enrichment analysis results of combined dataset low–high riskscore group genes. Table S7: GSVA analysis results of combined dataset high–low riskscore group genes. Table S8: mRNA–TF interaction network nodes. Table S9: mRNA–drug interaction network nodes. Table S10: mRNA–miRNA interaction network nodes. Table S11: mRNA–RBP interaction network nodes.

Supporting Information 2 Figure S1: GSEA, gene set enrichment analysis; GSVA, gene set variation analysis; Co-DEGs, common differentially expressed genes; ERSRGs, endoplasmic reticulum stress related genes; ERSRDEGs, endoplasmic reticulum stress related differentially expressed genes; GO, Gene Ontology; KEGG, Kyoto Encyclopedia of Genes and Genomes; SVM, support vector machine; LASSO, least absolute shrinkage and selection operator; ssGSEA, single-sample gene-set enrichment analysis.

Supporting Information 3 Figure S2: (A) Box plot of GSE7429 sample before normalization. (B) Box plot of GSE7429 sample after normalization. (C) Box plot of the combined dataset samples before standardization. (D) Box plot of the standardized combined dataset samples. (E) PCA plot of the combined dataset before batch processing. (F) PCA plot of the combined dataset after debatch processing. PCA, principal component analysis.

Supporting Information 4 Figure S3: (A–E) KEGG enrichment analysis results of ERSRDEGs. Visual display of pathway diagram: Chemical carcinogene-sis-receptor activation (A), pathogenic *Escherichia coli* infection (B), hepatitis C (C), autophagy-animal (D), and apoptosis (E). KEGG, Kyoto Encyclopedia of Genes and Genomes; ERSRDEGs, endoplasmic reticulum stress related differentially expressed genes.

Supporting Information 5 Figure S4: (A) Nomogram of model genes in the osteoporosis diagnostic model based on the combined dataset. (B) DCA diagram of the osteoporosis diagnostic model based on model genes in the combined GEO dataset. (C) ROC curve of the risk score of the com-bined dataset osteoporosis diagnostic model. (D) ROC curve of the risk score of the GSE7429 osteoporosis diagnostic model. DCA, decision curve analysis; ROC, receiver operating characteristic; AUC, area under the curve. The model has higher accuracy when AUC is above 0.9, the model has certain accuracy when AUC is between 0.7 and 0.9, and the model has lower accuracy when AUC is between 0.5 and 0.7.

Supporting Information 6 Figure S5: (A) A volcano plot showing the results of differential gene expression analysis between high- and low-risk groups in the combined GEO dataset. (B) GSEA of the combined dataset shows an enrichment plot of six biological functions. (C–H) GSEA shows that ERSRDEGs are significantly enriched in: IL2-PI3K pathway (C), photodynamic therapy-induced UPR (D), IL-18 signaling pathway (E), unfolded protein response (F), intra-golgi and retrograde golgi-to-ER traffic (G), and golgi-to-ER retrograde transport (H). ERSRDEGs, endoplasmic reticulum stress-related differentially expressed genes; GSEA, gene set enrichment analysis. The screening criterion for GSEA is *p* < 0.05.

Supporting Information 7 Figure S6: (A) Group comparison diagram and (B) heat map of GSVA results between high-risk and low-risk group samples in the combined dataset. GSVA, gene set variation analysis. *⁣*^*∗*^ represents *p* < 0.05; *⁣*^*∗∗*^ represents *p* < 0.01, which is highly statistically significant; *⁣*^*∗∗∗*^ represents *p* < 0.001, which is extremely statistically significant. GSVA screening was performed using p < 0.05 and log FC ranking to identify the top 10 positively and negatively enriched pathways. In the heat map, blue represents the low-risk group samples, while orange represents the high-risk group samples in the combined dataset.

Supporting Information 8 Figure S7: (A) C1QBP, (B) CES2, (C) CYB5R4, (D) RAB1B, (E) SERP1, (F) RNF13, and (G) UFSP2. The AlphaFold website generates per residue confidence scores (pLDDT) ranging from 0 to 100. Regions with pLDDT scores below 50 might represent isolated un-structured areas. When pLDDT < 50 (red regions), the model confidence is very low; when 50 < pLDDT < 70 (yellow regions), the model confidence is low; when 70 < pLDDT < 90 (light blue regions), the model confidence is moderate; when pLDDT > 90 (blue regions), the model confidence is very high.
