# Supplementary material for: Identification of Endoplasmic Reticulum Stress-Related Genes in Osteoporosis Pathogenesis
Source: Mediators Inflamm. 2025 Aug 30;2025:6726771. doi: 10.1155/mi/6726771 (PMC12413945; doi:10.1155/mi/6726771)
Supplement: Supporting Information 1 — Table S1: Osteoporosis datasets information list. Table S2: List of ERSRGs. Table S3: GSEA enrichment analysis results of combined dataset control-osteoporosis group genes. Table S4: GSVA enrichment analysis results of combined dataset control-osteoporosis group genes. Table S5: GO and KEGG enrichment analysis results. Table S6: GSEA enrichment analysis results of combined dataset low–high riskscore group genes. Table S7: GSVA analysis results of combined dataset high–low riskscore group genes. Table S8: mRNA–TF interaction network nodes. Table S9: mRNA–drug interaction network nodes. Table S10: mRNA–miRNA interaction network nodes. Table S11: mRNA–RBP interaction network nodes. [file 6726771.f1.doc]

Supplementary Table

Supplementary Table S1. Osteoporosis Datasets Information list.

|  | GSE56815 | GSE230665 | GSE7429 |
| --- | --- | --- | --- |
| Platform | GPL96 | GPL10332 | GPL96 |
| Species | Homo sapiens | Homo sapiens | Homo sapiens |
| Experiment type | Expression profiling by array | Expression profiling by array | Expression profiling by array |
| Tissue | monocytes | monocytes | B cells |
| Samples in Control group | 20 | 3 | 10 |
| Samples in Osteoporosis group | 20 | 12 | 10 |
| Reference | A novel approach for correction of crosstalk effects in pathway analysis and its application in osteoporosis research | Comparisons of gene expression between peripheral blood mononuclear cells and bone tissue in osteoporosis | In vivo genome-wide expression study on human circulating B cells suggests a novel ESR1 and MAPK3 network for postmenopausal osteoporosis |

Supplementary Table S2. List of ERSRGs.

| Gene Symbol | Gene Symbol | Gene Symbol | Gene Symbol | Gene Symbol | Gene Symbol | Gene Symbol |
| --- | --- | --- | --- | --- | --- | --- |
| HSPA5 | POMT2 | GBA2 | NOTCH3 | PLK1 | TACC3 | TUT1 |
| DDIT3 | INHBE | KLB | VIM | PRKAR1A | TARS1 | TXNDC17 |
| HERPUD1 | E2F1 | NOL3 | KCNH2 | RPS6KA1 | TCOF1 | U2SURP |
| EIF2AK3 | GH1 | RYR3 | PKM | SHH | TPR | USP39 |
| ERN1 | UBE2J1 | UPF1 | TNFSF11 | TFRC | TUBB6 | WDR82 |
| ATF6 | FBXO6 | CD109 | UBA1 | TGFB2 | UBQLN2 | YTHDF3 |
| ATF4 | SDF2 | COLEC10 | ENO1 | APC | UCP1 | ZBTB38 |
| XBP1 | KAT2A | DIO2 | CETP | ATP1A1 | ACTL6A | ATP5MG |
| EIF2S1 | CREBZF | LRP1B | PINK1 | B2M | ALDH9A1 | C1GALT1C1 |
| CASP4 | HSPB1 | MED25 | PRDX2 | DNM1 | ALG2 | CEMIP2 |
| PPP1R15A | HIF1A | MSI1 | SPHK1 | GSTP1 | ARFGAP1 | CIP2A |
| UFL1 | RTN1 | MSI2 | LGR5 | IFNAR2 | ASGR2 | EMC8 |
| ATF3 | RHBDD1 | MTDH | SPINK1 | KAT5 | ATG3 | ETV2 |
| HSP90B1 | BAD | NUDT5 | AHSA1 | NR5A1 | ATG4A | GCN1 |
| CASP3 | NPHS1 | PHLPP1 | WASF3 | PPP2CA | ATG4B | H1-6 |
| BCL2 | HFE | PHLPP2 | APLN | RARA | ATG9A | KCTD12 |
| MAP3K5 | FKRP | REG1A | PSMB8 | RHOA | ATP5MC3 | LRRC59 |
| CEBPB | NOS3 | SND1 | TCF7L2 | ACACA | ATP6V1F | LTV1 |
| MAPK8 | HSF1 | SSR2 | TRIM25 | ACTN1 | B4GALT7 | MCMBP |
| NFE2L2 | MAP1LC3B | TSC22D3 | VDAC2 | ALDOA | BACE2 | OCIAD2 |
| SCAMP5 | MSTN | CPEB4 | UBXN4 | CD44 | BBS4 | ORAI3 |
| TMED4 | PTPA | EBI3 | G6PC3 | CSNK1A1 | BICD2 | PEF1 |
| CREB3 | MUC5AC | ELOB | DAPK1 | CUL3 | BPNT2 | PSPC1 |
| DNAJC3 | IGF1R | FUT4 | LIPA | CYBB | CAP1 | QPCTL |
| TP53 | IGF1 | GPR4 | PLA2G6 | ESR2 | CAPZA1 | RPL36A |
| SYVN1 | SGK1 | HOXB9 | SIRT3 | GPHN | CCT7 | SLFN11 |
| NOD2 | TNFRSF10A | IL23A | GANAB | GSK3A | COIL | SPTSSA |
| CALR | APAF1 | IL32 | ATP13A2 | HPRT1 | CPSF1 | SRP9 |
| ATF6B | NR1H2 | IL33 | RUVBL2 | HSP90AB1 | CSE1L | STX17 |
| P4HB | DAB2IP | MIA2 | SEMA4A | MAPK9 | DAD1 | TEX10 |
| SESN2 | HSPA1A | MSRB1 | STIP1 | MUC1 | DDX39B | WBP1 |
| MBTPS2 | RAB1A | SEC61B | SLC39A7 | NCAM1 | DEK | XXYLT1 |
| TNFRSF10B | CHAC1 | STARD7 | TFG | PIK3C3 | DHX30 | YIPF3 |
| DDRGK1 | TLR2 | TSLP | PRDX4 | POR | DSG4 | ZDHHC1 |
| MANF | FOS | XAF1 | SIRT7 | PPP2R1A | EEF1D | CFAP20 |
| CXCL8 | ABCG2 | HRK | GABARAPL1 | ROR2 | EIF1AX | DHRS7 |
| DNAJC10 | CEBPA | MIOX | RPL30 | RPS19 | ENAH | DNAJB14 |
| VCP | SEC63 | RNF114 | UGGT1 | RRM1 | ETS2 | EPM2AIP1 |
| QRICH1 | CCDC88A | SF3A2 | COG5 | RXRA | FAR1 | FOXJ2 |
| DERL1 | NELL2 | SGMS2 | CLCC1 | TLR8 | FBXO11 | KLF16 |
| RNF186 | TULP1 | WTAP | SDF4 | TOP1 | GSPT1 | PCNP |
| MBTPS1 | ENAM | ART1 | ZFYVE27 | TPM1 | HDLBP | PHLDA3 |
| DERL2 | DNAJC12 | ATAT1 | JAGN1 | TUBG1 | HKDC1 | PRRC2A |
| INS | SERP1 | BLOC1S1 | RNFT1 | VLDLR | HMGCS1 | RABL6 |
| WFS1 | TMEM241 | DNAJB12 | USF3 | VWF | HNRNPF | RRS1 |
| CASP12 | STUB1 | LECT2 | SMIM14 | YWHAG | KIFC1 | SRXN1 |
| PRKN | SLC38A2 | NOXA1 | PIGBOS1 | ACTA1 | KRT2 | SYNRG |
| SIRT1 | CTH | OMA1 | CAT | AK2 | LARP7 | TEX2 |
| FICD | ATM | S100G | GRIN1 | ALDH1A1 | LIN28A | TIPRL |
| HMGB1 | OTUB1 | SLC30A3 | HDAC1 | BRD4 | MAGED1 | TOR1AIP2 |
| CREB3L1 | IFNA1 | YTHDF1 | HSPD1 | CD8A | MAGT1 | TRIM59 |
| CASP9 | KRAS | H2BC3 | IDE | CSF3R | MAPK8IP3 | ZNF622 |
| TMEM117 | GORASP2 | IRGM | ATF2 | CTSC | NR0B2 | ZNF91 |
| PTPN1 | NR3C1 | RHBDL3 | YWHAB | CYLD | NUCB1 | FCF1 |
| RNF183 | TUBA1B | TMEM100 | KRT14 | DGAT1 | P3H1 | PRRC2C |
| BBC3 | UFD1 | C1QTNF9 | ETFA | DHCR7 | PA2G4 | TTC27 |
| TMBIM6 | NDUFS4 | DELE1 | ATG5 | FTO | PCBP2 | FOXO6 |
| ERO1A | NNAT | H2AC4 | DHCR24 | HSPA9 | PIGT | HDGFL2 |
| RTN3 | THBS1 | RIC3 | NR5A2 | MAN1B1 | PLD3 | OSTN |
| DNAJB9 | ERP44 | USP35 | PLN | MAP4K4 | PLIN3 | SREK1IP1 |
| BAX | TRAF6 | BATF2 | DHX9 | MIF | PLS3 | STYXL1 |
| EIF2AK2 | SOCS3 | GPR78 | EDEM1 | MPL | PPA1 | TMEM176B |
| CREB3L3 | TRAP1 | SSUH2 | NUDT1 | MSN | PSMB2 | YIPF4 |
| EIF4G1 | TXN | PML | DDIT4 | NR1H4 | PSMB6 | EMC7 |
| HYOU1 | BHMT | PPP2CB | HNRNPC | PCYT1A | PSMC4 | POTEE |
| CASP8 | AGT | RASGRF1 | LRBA | PDK1 | RAB10 | QNG1 |
| CCL2 | YWHAE | RASGRF2 | NINJ1 | PLK4 | RAB3GAP1 | SYS1 |
| PRNP | RAC1 | NRBF2 | OLA1 | PRKCI | RPS13 | H2AC21 |
| TMEM214 | FUS | BDNF | PITRM1 | TMPRSS2 | RPS16 | IRGQ |
| TRIB3 | ERLIN2 | PRKRA | MAN2C1 | TRPV6 | SCYL1 | MAP1LC3C |
| BAG6 | TFEB | GAPDH | PGAM5 | AK1 | SIL1 | HNRNPA1L2 |
| PDIA3 | OPTN | ACTB | ERVW-1 | AKR1C3 | SLC30A10 | ZNF99 |
| DDX3X | MYOC | CAD | FNDC3B | ALDH3A2 | SLC30A2 | NME1-NME2 |
| CANX | UBQLN1 | EPHA3 | SVIP | APRT | SRSF2 | POTEF |
| JUN | CYB5R4 | HNRNPA2B1 | EPHB4 | ARHGDIA | SRSF9 | RESP18 |
| ANKS4B | CALHM1 | ELAVL1 | AR | ATP5F1A | SUCLG2 | IFNL4 |
| SLC39A14 | PHLDA1 | RPL19 | HDAC2 | CD81 | TBCB | ARMCX5-GPRASP2 |
| SELENOS | ZFAND2B | STK38 | PPP3CA | CDH5 | TMEM43 | CD40 |
| TRAF2 | RETREG3 | RGS16 | CBL | CFL1 | TRDMT1 | WNT5A |
| NUPR1 | RNF121 | RPL23 | NPM1 | CRKL | TRIP13 | GABRG2 |
| NLRP3 | PARK7 | VIL1 | PRKCA | CSTB | USP25 | TAOK1 |
| PPP1CA | TUBB | UBR4 | PRKDC | CYBA | UVRAG | CYP2B6 |
| DNAJB11 | NDRG1 | ILF2 | CAPN1 | DDB1 | ZMPSTE24 | TOLLIP |
| ATP2A2 | PPARD | CHERP | LEPR | EEF1A2 | ACBD3 | EVC |
| UMOD | SP1 | TANGO2 | PGK1 | EZR | AIFM2 | SEPTIN5 |
| CASP7 | VDAC1 | COMP | ATP1B1 | FGF23 | ATE1 | SMAD3 |
| PDIA4 | EEF1A1 | COL4A2 | GGT1 | FTL | ATG12 | GRIN2A |
| ERP29 | CST3 | ARCN1 | USP9X | GJB1 | C9orf72 | GRIN2B |
| OS9 | RPS10 | CDK6 | WT1 | GPC3 | CBY1 | PIK3CA |
| BCL2L11 | TRPM2 | TLR3 | ABCA1 | GPD2 | CCDC6 | COL1A1 |
| CYCS | HRC | CASP1 | ABCC6 | HADHA | CCT6A | FBP1 |
| PPP1CC | RPS27A | PGR | DUSP6 | HADHB | CXCL5 | GSR |
| BOK | MAP2K1 | CASP6 | FGF1 | HSD17B10 | EDEM2 | RHEB |
| CCDC88B | IL10 | FAH | GRN | HSD17B4 | EEF1G | SLC6A3 |
| DERL3 | STAR | TRPV1 | NGFR | KDM4B | EIF3E | VDR |
| TGFB1 | RAB6A | CCND3 | PRKAB1 | KRT5 | EIF3H | BRCA2 |
| ASNS | GRP | CNR1 | SCARB1 | LMNB1 | FBXO32 | HDAC5 |
| TXNDC12 | NPLOC4 | CCNA2 | TPI1 | MYH6 | FFAR4 | NBN |
| NCK1 | OSBPL5 | MAN1A2 | AKR1B1 | PEBP1 | FOXD3 | CDH3 |
| SIGMAR1 | PDX1 | H2AX | EHMT2 | PKD1 | GNPDA1 | DCN |
| NOD1 | PIK3CG | LGALS1 | FADD | PLOD3 | GSTK1 | DLG4 |
| SGPP2 | IGF2BP2 | INHA | HK2 | PSAT1 | H1-5 | GCH1 |
| GSK3B | BIRC3 | IGF2BP3 | HNF1A | REL | HSPA13 | EIF4EBP1 |
| RETREG1 | STARD4 | TNFRSF12A | LBR | RFC1 | IRS4 | MAP4K2 |
| CREB3L2 | MMP9 | ID1 | LDHB | RIPK2 | KCNIP1 | MMUT |
| PPP1CB | ALK | KLF15 | CBLB | ROCK2 | KLRK1 | PITX2 |
| SERPINA1 | SRC | ILF3 | FGF2 | RPSA | MAGI1 | HP |
| TMEM33 | RPL11 | RBM3 | SIRPA | SGPL1 | MYL6 | SERPINF1 |
| MTOR | ACLY | VMA21 | TWIST1 | SHMT2 | NAT10 | NR1I3 |
| NHLRC1 | COL4A1 | GZMK | ALOX12 | STT3A | NBAS | PON3 |
| SREBF1 | EPHX2 | MAP1LC3A | ASL | SUMO1 | NCEH1 | PRDM1 |
| AIFM1 | F7 | CTNNB1 | ATP2C1 | TMPO | NOLC1 | SLC1A4 |
| AKT1 | KCNN4 | HLA-B | BGN | TP63 | NSMAF | UBB |
| TLR4 | TRPA1 | LPCAT3 | DKK1 | UQCRFS1 | NUCB2 | ABCA7 |
| PARP1 | AHR | UCHL1 | HSD11B2 | WRN | PDCL | CLOCK |
| APP | BMP2 | OSGEP | LAMP2 | YWHAH | PEX13 | CXCR1 |
| CREBRF | ARRB1 | TP53RK | PLK2 | A2M | PIEZO1 | DISC1 |
| UBA5 | CD274 | TPRKB | PSMA7 | AGXT | PIGS | DNASE1 |
| ATP2A3 | PSMD4 | LAGE3 | RAD23B | AIMP1 | PITPNB | DRD3 |
| SEL1L | RTN4 | ULK1 | SMURF2 | AKR1C2 | POP1 | IL7 |
| CLU | SET | DNM1L | TNFSF13 | ALG1 | PPA2 | KLF6 |
| CDK5RAP3 | SLC8A1 | SEC23B | TNFSF13B | CDC20 | PSMB3 | NNT |
| HERPUD2 | CES2 | SEC61A1 | APLNR | CGA | PSME3 | SBDS |
| ERN2 | HAMP | CHEK2 | EIF2B1 | CLTC | RAB14 | SEPSECS |
| UFM1 | IL22 | RAF1 | NCL | COL6A3 | RAB1B | SOD3 |
| SDF2L1 | PTX3 | MDM2 | PSMB5 | DUSP1 | RAB3GAP2 | SRF |
| MAPK1 | EIF2B3 | PIK3CD | RPL18 | FDFT1 | RARRES1 | HNRNPD |
| PTGS2 | FAS | BCR | SENP1 | GCLC | RBM14 | IL15 |
| BRSK2 | TNFRSF1A | CBS | SHC1 | GFER | RECK | SECISBP2 |
| GRINA | PRPS1 | GJA1 | UBC | GJB2 | RPA3 | AGRP |
| BSCL2 | PRPS2 | PRKCG | CFP | GNAI3 | RPL27A | GPR39 |
| HMOX1 | SEPTIN7 | TF | INPP5K | ITPR3 | RPL34 | PSMC5 |
| TUG1 | INSR | DNM2 | MICU1 | KHK | RPLP1 | RFK |
| NFYA | PSEN2 | MMP3 | POSTN | MDH1 | SENP3 | TBC1D1 |
| BCAP31 | ANXA5 | RPS6KB1 | RPL13 | MYH10 | SF1 | VAMP7 |
| SOD1 | ATF1 | SLC12A2 | RPL8 | P4HA2 | SIRT4 | GORASP1 |
| HTT | DYSF | JUP | DEGS1 | PLOD2 | SLC25A22 | NFIC |
| TNFSF10 | SFTPA2 | RYR1 | JPH3 | PPIA | SNX3 | S100P |
| CASP2 | CD36 | TTN | PNPLA3 | PPP2R2B | SPECC1L | SCD5 |
| NIBAN1 | RIPK1 | WEE1 | RPL7 | SFN | SRP19 | TAOK3 |
| UFC1 | SPARC | IMPA1 | RPL7A | SNTA1 | SRSF3 | VPS13B |
| BCL2L1 | RYR2 | MAPK13 | SHBG | SUCLG1 | SRSF7 | CERS6 |
| NFKB1 | TGM2 | NQO1 | SRSF6 | TFPI | STC1 | SKAP2 |
| NCK2 | ERAP1 | TFAP2A | STING1 | TKT | SUMO2 | SHB |
| TTC23L | SRI | TNFAIP3 | UBE2S | TP73 | TAB3 | TSEN54 |
| PRKAA1 | CAV3 | TRAF3 | BAG2 | TUBB2A | TAGLN2 | CITED1 |
| C6orf120 | MATN3 | WNT1 | INSIG1 | WWOX | TEAD3 | SLC30A6 |
| SFTPC | RCN1 | EEF2K | MYBBP1A | AARS1 | TNFAIP6 | UACA |
| NPTX1 | TBL2 | IRF1 | PRPH2 | ABCB7 | TRPC1 | CEP85L |
| IL1B | CRELD2 | KLF4 | SAR1A | ACAT2 | TSN | CLEC4E |
| ABL1 | RRBP1 | P2RX7 | SERPINA12 | ADM | VPS45 | GPR85 |
| EIF2B5 | CDK4 | RUNX2 | TES | APPL1 | WDR45 | NFX1 |
| PDIA2 | DYRK1A | SLC2A4 | UCHL5 | ARRB2 | ADPGK | PLAGL2 |
| PDIA6 | GYS1 | SLC6A2 | ADRM1 | BSG | CAND1 | TOX3 |
| ITPR1 | FLNA | CHRNE | CD68 | C1QBP | COPS8 | NT5DC1 |
| PMAIP1 | RPL5 | IL10RA | COPZ1 | CCT5 | CSDE1 | C1QTNF12 |
| AGR2 | ERCC6 | MEF2A | DBF4 | CHRNA7 | CTR9 | CTSD |
| TARDBP | DYRK1B | AHSG | DSPP | CKB | DDX3Y | SLC2A1 |
| VEGFA | NEDD4L | BMP6 | EIF5B | COASY | EIF3L | STAT1 |
| CFTR | IGFBP2 | CPOX | HNRNPM | CRBN | EIF3M | CXCR4 |
| MCL1 | CXCL1 | IL12A | MBOAT7 | CXCL10 | FUBP1 | HRAS |
| TMTC3 | EIF4B | IL12B | QSOX1 | DFFA | FYCO1 | ALPL |
| IGFBP1 | PCSK6 | POLR1C | RPL6 | DVL2 | GPAT3 | G6PD |
| NFYC | WDR77 | POMT1 | CLIC4 | ECHS1 | GRPEL1 | LDHA |
| LCN2 | OPA1 | TIMP1 | INSIG2 | EGLN2 | IST1 | TH |
| PSEN1 | SEC16A | TRRAP | MAP1A | EPHX1 | MAP4 | TSC2 |
| BIRC2 | TMEM258 | ACO1 | MICA | GALNT2 | NCR1 | C3 |
| TMEM259 | TMEM238L | CALCA | NDRG2 | GPX1 | NDUFAF3 | CHUK |
| LRRK2 | RPL26 | ENDOG | SP2 | GSTO1 | PSCA | GLUD1 |
| KEAP1 | RPN1 | H6PD | COL22A1 | HARS1 | PSMC6 | HDAC3 |
| NR4A1 | AURKA | KRT19 | GADD45GIP1 | HLA-A | PSMD1 | IRAK4 |
| SREBF2 | FGFR4 | ATXN7 | KLF9 | HPN | PSMD6 | TRPC6 |
| TERT | APOA1 | FOSL2 | RNF182 | HPSE | PTGR1 | CES1 |
| EIF2AK4 | CTSB | NPHS2 | TXNDC11 | KLK4 | RABEP1 | HDAC9 |
| ORMDL3 | FLT1 | PPP2R5C | CCDC170 | LETM1 | RPL28 | IGF2 |
| STAT3 | IL6ST | RBP3 | RNF152 | LGALS3 | RPS11 | LRP1 |
| EGFR | ARG1 | UBE2G1 | TTLL6 | LRPAP1 | RPS15 | MAP2K3 |
| FABP4 | GDNF | UBE3C | MAGEH1 | MAP1B | S100A7 | NF2 |
| YIPF5 | MMP13 | CDIPT | CDKN2A | MAP3K14 | SACM1L | NRG1 |
| RHO | RUNX1 | CDKN3 | EPAS1 | MAPRE1 | SAFB | PROS1 |
| CHAC2 | USP7 | CHM | STAT6 | MTHFD1 | SNRNP70 | SLC25A4 |
| HDAC6 | ACE2 | DAP3 | ASS1 | NCOA1 | STRAP | GRK2 |
| PPARG | ANGPT2 | ELOC | GLA | NCOA2 | TLR10 | KCNJ5 |
| IAPP | CHRM3 | FABP5 | ROCK1 | NME1 | TMEM165 | MAP2K4 |
| MAPK3 | EEF2 | RBM8A | GLB1 | NONO | UBA52 | MC1R |
| PPP1R15B | LYN | TAF6 | RAB7A | NPY | WNT16 | PDCD1 |
| AGER | GNAI1 | CHMP1A | SPTAN1 | PABPN1 | ZNF331 | PLA2G4A |
| DPAGT1 | IRS1 | ELAVL2 | TLR7 | PIK3R4 | ABCE1 | POLG |
| EIF2A | RPA1 | GLRX3 | TUBB3 | POFUT1 | AKAP11 | POMC |
| IFNG | CCN2 | MOV10 | ANXA2 | PPP2R2A | AKAP8 | TGFB3 |
| GPR37 | ADAMTS5 | MRPS28 | CARM1 | PRDX3 | ALYREF | VCL |
| BAK1 | CYP1A2 | PSMD10 | CPE | PSMA3 | ANKFY1 | YAP1 |
| MAGEA3 | KAT6B | SERPINB7 | FURIN | PSMA6 | AP3M1 | ABCC8 |
| CDKN1A | CYP2C19 | TAF2 | MDH2 | PSMB1 | ATG2A | ATP7A |
| SCD | GADD45A | TAF5L | MYH9 | RBBP4 | BABAM1 | CYP2A6 |
| PIK3R1 | NR1I2 | UBE2J2 | NCOA3 | RPL35 | BCL2L10 | DKC1 |
| PPARGC1A | SOAT1 | CXCL2 | PIN1 | SIRT6 | CCL8 | GALT |
| LEP | OLR1 | HSPB2 | PTGER2 | SLC27A4 | CLPTM1L | GUCY2C |
| TXNIP | TRPV3 | KDELR1 | SPTLC2 | SLC39A8 | DPH5 | HLA-DRB1 |
| SPTLC1 | IL11 | KLRG1 | UBE2I | SLC7A11 | EHD4 | HMGCR |
| CFLAR | CCN1 | MRPL12 | VHL | SMARCC2 | EIF3B | KCNJ11 |
| HSP90AA1 | SEPTIN4 | PDCD6 | WNT3A | STK3 | EIF3C | PRF1 |
| SQSTM1 | HCRT | TAF10 | YES1 | TBX21 | ENC1 | SLC40A1 |
| APOE | UTS2 | TAF12 | YWHAZ | TFE3 | EOGT | TBX2 |
| CDNF | RCE1 | USP34 | GFPT1 | UBE2D1 | EPG5 | CDK5R1 |
| LMNA | IFI6 | DNAJA2 | GLP1R | UBE2D3 | ESYT1 | CLPP |
| MAPK14 | SELENOP | FAM120A | LYZ | UGT1A9 | GINS2 | CS |
| EPM2A | ULBP1 | GIMAP5 | MAPKAPK2 | USP5 | GPAT4 | CXCL12 |
| RELA | TSPYL5 | MRPS9 | MYH11 | ADNP | H3C1 | GRK3 |
| FGF21 | NR1H3 | POMGNT2 | OXTR | AKR1C1 | HEY2 | GRM3 |
| NOX4 | FLOT1 | SF3B3 | PFN1 | ALDH1B1 | HM13 | HNRNPK |
| ERBB2 | EZH2 | TAF9 | YWHAQ | ANP32A | HSPA1B | IRF3 |
| TNF | STK11 | AP1G2 | AP3B1 | APBB1 | JAZF1 | PCNT |
| NOS2 | ALB | CASC3 | ATP6V1A | ATG7 | LMO7 | PDE4B |
| MFN2 | PRDX1 | G6PC2 | LONP1 | ATXN2 | LRRFIP2 | RBP4 |
| EIF1 | TBK1 | MAGOH | MYH14 | CASP5 | LYAR | SLC31A1 |
| ADIPOQ | LPIN1 | MRPL1 | SOX2 | CCL5 | METTL14 | SQLE |
| MX1 | FAP | MRPS18B | SPP1 | CDA | MPRIP | STMN1 |
| FAM20C | HTRA2 | NCBP2 | USP15 | CHMP4B | MRPL11 | TLR9 |
| SNCA | TRPC3 | SUPT3H | AKR1A1 | CNBP | MUC2 | TUFM |
| HSPA4 | HTRA1 | TAF5 | ATP6AP2 | CNP | MYL6B | XRCC5 |
| PROC | IRS2 | TAF8 | CHI3L1 | CRH | MYO1D | AKR1B10 |
| COL4A3 | PFKFB3 | MRPS6 | COL6A2 | CUL4B | MYRF | ALPP |
| EXOSC9 | SMURF1 | RALYL | EMD | DAXX | NACA | ALS2 |
| EXOSC8 | CUL1 | RO60 | ENTPD5 | DBT | NIPSNAP2 | CRTC1 |
| PPP1R8 | RUNX3 | SERPINB4 | FKBP4 | DDX41 | NOP58 | CSTA |
| PON2 | ABCG1 | TADA3 | GALC | DLG1 | NUS1 | DIAPH3 |
| PTPN2 | CAPN5 | TAF6L | GLO1 | DMP1 | PNO1 | DLX5 |
| THBS4 | CDC27 | TMEM94 | HSPA1L | DROSHA | PREB | EIF2B2 |
| PPARA | SSB | ENY2 | RPL15 | EBP | PSMG2 | ELOVL5 |
| MSRB3 | CEBPD | H4C16 | RUVBL1 | EIF3A | RAB39B | GAK |
| TMEM208 | ERAP2 | MRPS31 | SLC1A5 | EIF3F | RAI14 | HAX1 |
| YY1 | USP22 | NFKBIZ | TG | EPRS1 | RASSF5 | HIPK2 |
| HSPA8 | SAA1 | SF3B5 | COPB2 | ESD | RBM39 | HNRNPU |
| MAPT | ERGIC3 | TADA2B | CXCR3 | FKBP8 | RHBDF1 | IRF4 |
| FOXO1 | H1-1 | WDR74 | EIF2B4 | FLII | RIF1 | MYO7A |
| PLP1 | MARCHF5 | ATXN7L3 | RPS6 | GAD2 | RINT1 | PSMD14 |
| BID | PDIA5 | KIF27 | SFPQ | GALE | RNF213 | SERPINB5 |
| BECN1 | FKBP9 | PTCD1 | SLC33A1 | GBF1 | SCYL2 | SKP1 |
| EIF2AK1 | FNDC5 | RPL39 | SPOP | HLA-C | SF3A3 | TAB1 |
| RNF13 | SGF29 | TADA1 | ATG16L1 | HMGA2 | SMPD4 | TRADD |
| TOR1A | ERP27 | UBR3 | CCT2 | IL17A | SNRPD2 | ANXA3 |
| GAA | TMX1 | ZACN | PSMD2 | KPNB1 | SYNCRIP | DGAT2 |
| SLC37A4 | GBA1 | KLHDC10 | TCP1 | KRT10 | TOE1 | DLG3 |
| PRKAA2 | JAK2 | MRPL52 | TMEM38B | LAMP1 | TOMM70 | EREG |
| CAPN2 | STAT5A | SUPT7L | ATAD3A | MCCC2 | ZC3HAV1 | H1-4 |
| ZC3H12A | CISH | BCL2L15 | COPB1 | MLXIPL | ARL8B | HAS2 |
| AQP11 | CPA1 | CDKN2AIPNL | CPB1 | MMP17 | BYSL | KCNE2 |
| CAMK2B | PTEN | CDV3 | DEPTOR | MTMR14 | C1QTNF3 | MGAM |
| AGR3 | F2 | DDX60L | GABARAPL2 | MYO6 | CACYBP | PRPF8 |
| BNIP1 | BACE1 | ZCCHC3 | GPX7 | NMT1 | CEMIP | RPL3 |
| LACC1 | MTHFR | FLYWCH2 | HNRNPR | NOP56 | CIRBP | SLC7A1 |
| TUSC3 | IL1R1 | C3orf70 | IRAK2 | NUDC | ERLEC1 | SLC8A3 |
| PNLIP | LMAN1 | MSBP2 | RPL10A | P4HA1 | GATAD2A | APEH |
| TTF2 | PURA | PRSS1 | SLC30A8 | PDCD4 | GLOD4 | BACH1 |
| CTRC | IFNB1 | IL6 | ATG13 | PDGFA | GLT8D1 | CAPNS1 |
| CAMK2G | CARD14 | CYP2E1 | BAG5 | PGAM1 | GNL2 | CAVIN1 |
| ESR1 | ACSL3 | VAPB | CCT8 | PICALM | H1-3 | FLAD1 |
| AGTR1 | EIF2S2 | PAWR | COPG1 | PPIF | HNRNPAB | GHRL |
| DIABLO | STARD5 | PIK3R2 | FGL1 | PTRH2 | HNRNPUL1 | HAS3 |
| APOL1 | SUPT20H | ALOX5 | NMNAT2 | RACK1 | HTATSF1 | PIGR |
| MGAT5 | SUPT20HL1 | ALOX15 | PSMD8 | RAMP2 | IER3 | POLR3K |
| SEC62 | FASN | MAP3K20 | RCN2 | RPS20 | KRT23 | PRDM2 |
| SNCG | CNGA3 | KCNJ8 | REEP5 | RPS27 | MRPL19 | RPL4 |
| MET | IGF2BP1 | AFF4 | RPL24 | S100A9 | MRPL39 | RRAGC |
| LDLR | MSBP1 | STC2 | RPLP2 | SAE1 | MT1E | SCAP |
| HNF4A | ATP2A1 | CERT1 | RPS2 | SEC31A | MUL1 | SLC45A2 |
| PRMT1 | FCGR2B | ELAVL4 | SLC39A13 | SH3GL1 | NAPA | SLC8A2 |
| FOXA2 | CCND1 | MARCKS | UBE2G2 | SMARCC1 | NCLN | SMG1 |
| VCAM1 | CREBBP | SERINC3 | CAMP | SPHK2 | PHPT1 | STK25 |
| EIF5A | RPS6KA3 | SRPX | GDI2 | TAPBP | PLP2 | AGXT2 |
| RAB9B | PRKCD | USP19 | IBTK | TREH | PSMG1 | BHLHE40 |
| BRAF | SMAD2 | TMED2 | RPS28 | TUBB4B | RAB21 | BNIP3 |
| INS-IGF2 | CDK2 | SELENOK | UFSP2 | UBTF | RNH1 | CLSPN |
| GDF15 | HSD11B1 | LOC122513141 | WLS | USP10 | RPS18 | COLEC12 |
| CREB1 | ALDH2 | CDK5 | ANP32B | AHCYL1 | RPS21 | FLOT2 |
| PCK2 | CDH1 | CEL | BST2 | ANXA7 | RRP1 | H1-2 |
| FGF19 | NTRK1 | PPIB | EIF3D | ASPH | RTCA | JDP2 |
| S100A16 | SERPINC1 | KL | GPX8 | BACH2 | SAFB2 | NBR1 |
| TRAM1 | ABCC1 | SERPINH1 | SF3A1 | BAG3 | SLC15A4 | NMI |
| GTF2I | CDKN1B | AMFR | EMC1 | BANF1 | SRRM1 | RPL12 |
| STIM1 | GFAP | RPLP0 | IPO5 | CALB1 | SSR3 | RSAD2 |
| XIAP | IFNAR1 | BIRC5 | NDFIP1 | CALU | STAU1 | RUBCN |
| PCSK9 | MYB | TXNDC5 | SAMD8 | CBX3 | SUB1 | SERPINB1 |
| PPT1 | SMPD1 | ATL3 | SELENON | CCL20 | TCF25 | SLC4A11 |
| IL24 | AFP | SRP54 | COPS6 | CCL7 | TIMM13 | STIM2 |
| SLC30A5 | CTCF | COPA | MYDGF | CD63 | TRIM13 | DDX39A |
| PARN | FBN1 | POMP | RNF185 | CHST6 | UQCR10 | MGRN1 |
| PPP1R12A | KAT2B | TEX264 | RPAP2 | CRTAP | USO1 | NLRX1 |
| EXOSC3 | NAMPT | ATF5 | TM9SF4 | DHDDS | WBP11 | RPL14 |
| PPP1R3A | RAB27A | EP300 | ZDHHC6 | DUSP10 | ZNF638 | BIK |
| DIS3 | TSC1 | ELANE | ASB7 | EIF5 | ABHD10 | DDX21 |
| EXOSC2 | ROS1 | TPT1 | FAM3A | ERCC8 | APMAP | DYNC1LI2 |
| EXOSC5 | UCP2 | PNLIPRP2 | TM7SF3 | ERLIN1 | ARFGAP2 | FCRL5 |
| KHSRP | DDX5 | UBE2D2 | ERMP1 | FADS1 | ATP5ME | GALNT6 |
| EXOSC1 | IL2 | UBE4B | RNF19B | GPAA1 | AUP1 | IL37 |
| PPP1R10 | MINPP1 | COL10A1 | CGRRF1 | GZMA | BPNT1 | LAMP3 |
| PPP1R14A | OGT | SLC22A13 | TMEM131 | HEY1 | BRIX1 | RTCB |
| PPP1R1A | ANG | VMP1 | HEATR3 | IDI1 | BZW1 | SUCNR1 |
| PPP1R2 | APOH | NFE2L1 | MT-ATP6 | KCNN2 | CCPG1 | CALCB |
| PPP1R9A | ARID1A | TRB | BRI3BP | KLC1 | CDC42EP1 | CAVIN2 |
| EXOSC7 | DNAJB1 | MYC | IER3IP1 | KPNA3 | ERGIC2 | CSMD1 |
| NFYB | HES1 | PCSK1 | MPZ | KRT16 | GLT8D2 | GIP |
| PPP1R3C | LARS2 | DDOST | ELN | LIN28B | H2AZ2 | GNLY |
| PPP1R7 | MTTP | ORAI1 | SNCAIP | MATR3 | IKBIP | IL1F10 |
| EXOSC4 | RDH12 | RPS3 | PCNA | MGST1 | LAPTM4A | IL36A |
| PPP1R13B | AREG | YBX1 | PON1 | MPST | LARP4 | KLF11 |
| CEBPG | CDC37 | OSBPL8 | UBE3A | MYO1C | LSG1 | PFDN1 |
| PPP1R14B | CHKA | CAV1 | CA4 | MYOCD | MAP1S | SEM1 |
| PPP1R14C | COX6B1 | KDR | PMM2 | NOX1 | MRPL2 | SGPP1 |
| PPP1R16B | DAPK3 | NOTCH1 | SIAH1 | NUP133 | NASP | SLITRK4 |
| PPP1R3F | FCN3 | CSNK2A1 | CLPB | PABPC1 | NTPCR | YTHDF2 |
| DCP2 | FOXM1 | PHB1 | SLK | PAFAH1B2 | PDZD8 | ATG2B |
| PPP1R1C | NLRP1 | TXNRD1 | TOM1 | PIGK | PFDN2 | FCRL4 |
| PPP1R3B | RBX1 | CLIC1 | ALG6 | PORCN | RBM25 | FCRL6 |
| EXOSC6 | SEC23A | RNF5 | DKK3 | PPIL1 | RETSAT | GPSM1 |
| PPP1R12C | CTBP2 | PCMT1 | MFSD2A | PSMA1 | RHBDD2 | SCARA3 |
| PPP1R3D | CUL5 | TGM6 | SIAH2 | PSMA5 | RPL22L1 | SMG8 |
| PPP1R14D | DUSP5 | BRCA1 | MAP2K2 | PTP4A1 | RRP12 | SYT13 |
| PPP1R3E | EPO | CORIN | ADAM10 | PUF60 | SART3 | UCN |
| PPP1R3G | METTL3 | NSFL1C | ATR | RANBP1 | SHQ1 | UXT |
| NRAS | PHB2 | FOXO3 | ERBB3 | RAP1GDS1 | SLIRP | ABT1 |
| COL2A1 | PIM2 | TUBB4A | IKBKB | RBMX | SNRNP40 | CGAS |
| EDN1 | PROKR2 | UBE2N | PTPN11 | RNF168 | SNRPA1 | FITM2 |
| CDK1 | ST3GAL4 | USP14 | IDH1 | RPN2 | SNRPD3 | PCDH20 |
| ERG | TAF4 | GABARAP | CAMK2A | RPS15A | SP8 | TSKU |
| HNRNPA1 | TIA1 | GNE | PRKACA | RSPO2 | SRP14 | ZXDC |
| EGR1 | ANKRD1 | CDKAL1 | ADAM17 | S100A8 | STAU2 | ADM2 |
| PTGES3 | BAG1 | ATG14 | CA2 | SEC24D | STX18 | DDI2 |
| TRIM21 | CAP2 | CCDC47 | COMT | SGK3 | TCF19 | OIP5 |
| TTR | CCT3 | CKAP4 | FN1 | SMARCA5 | TOR3A | ANGPTL8 |
| NGF | CCT4 | PARP16 | MAP3K7 | SNRPB | TRIM68 | SPRN |
| APOB | CRELD1 | HDAC4 | PHGDH | STK26 | TTLL12 | SPX |

ERSRGs,Endoplasmic reticulum stress Related Genes.

Supplementary Table S3. GSEA enrichment analysis results of Combined dataset Control- Osteoporosis group genes.

| ID | setSize | enrichmentScore | NES | pvalue | qvalue | rank |
| --- | --- | --- | --- | --- | --- | --- |
| BIOCARTA_IL2_PATHWAY | 22 | -0.57036 | -1.72397 | 0.011407 | 0.118645 | 1624 |
| BIOCARTA_IL5_PATHWAY | 10 | -0.72145 | -1.8015 | 0.005792 | 0.075579 | 926 |
| BIOCARTA_IL17_PATHWAY | 14 | -0.69596 | -1.88745 | 0.003876 | 0.064751 | 1567 |
| REACTOME_RHOBTB1_GTPASE_CYCLE | 21 | 0.701473 | 2.181367 | 0.002058 | 0.046486 | 1011 |
| WP_MITOCHONDRIAL_GENE_EXPRESSION | 16 | 0.642958 | 1.861247 | 0.00404 | 0.065807 | 1291 |
| WP_TYPE_I_COLLAGEN_SYNTHESIS_IN_THE_CONTEXT_OF_OSTEOGENESIS_IMPERFECTA | 27 | 0.499213 | 1.649603 | 0.012793 | 0.127389 | 2529 |

**Supplementary Table S4. GSVA enrichment analysis results of Combined dataset Control- Osteoporosis group genes.**

|  | logFC | AveExpr | t | P.Value | B |
| --- | --- | --- | --- | --- | --- |
| REACTOME_COMPETING_ENDOGENOUS_RNAS_CERNAS_REGULATE_PTEN_TRANSLATION | 0.932886 | 0.008701 | 4.429515 | 7.46E-05 | 1.332133 |
| REACTOME_HISTIDINE_CATABOLISM | 0.557615 | -0.03284 | 3.743419 | 0.000586 | -0.35538 |
| REACTOME_RUNX1_REGULATES_TRANSCRIPTION_OF_GENES_INVOLVED_IN_WNT_SIGNALING | 0.552371 | 0.029454 | 4.380546 | 8.67E-05 | 1.2091 |
| REACTOME_FREE_FATTY_ACID_RECEPTORS | 0.525847 | -0.01841 | 2.524361 | 0.015781 | -3.01931 |
| CLIMENT_BREAST_CANCER_COPY_NUMBER_DN | 0.518328 | -0.00551 | 3.974874 | 0.000296 | 0.203896 |
| NIKOLSKY_BREAST_CANCER_15Q26_AMPLICON | 0.486881 | -0.02288 | 4.192289 | 0.000154 | 0.739231 |
| LIU_IL13_MEMORY_MODEL_DN | 0.456748 | 0.032811 | 3.158484 | 0.003062 | -1.70362 |
| REACTOME_CONJUGATION_OF_BENZOATE_WITH_GLYCINE | 0.446638 | 0.018132 | 2.117978 | 0.040617 | -3.75643 |
| REACTOME_NEGATIVE_FEEDBACK_REGULATION_OF_MAPK_PATHWAY | 0.442991 | -0.01469 | 2.617725 | 0.012544 | -2.83728 |
| WEBER_METHYLATED_LCP_IN_FIBROBLAST_UP | 0.440711 | -0.02271 | 3.174269 | 0.002934 | -1.66871 |
| FARDIN_HYPOXIA_9 | -0.41421 | 0.006105 | -2.74139 | 0.009193 | -2.58952 |
| ITO_PTTG1_TARGETS_UP | -0.43354 | -0.01119 | -3.34418 | 0.001835 | -1.28735 |
| REACTOME_CHL1_INTERACTIONS | -0.43431 | -0.03621 | -3.46315 | 0.001313 | -1.01456 |
| REACTOME_CREB1_PHOSPHORYLATION_THROUGH_THE_ACTIVATION_OF_CAMKII_CAMKK_CAMKIV_CASCASDE | -0.44638 | -0.02628 | -3.54511 | 0.00104 | -0.82406 |
| MCBRYAN_TERMINAL_END_BUD_DN | -0.45078 | -0.01066 | -3.22725 | 0.002537 | -1.5509 |
| REACTOME_SUMO_IS_CONJUGATED_TO_E1_UBA2_SAE1 | -0.4547 | 0.029635 | -2.46291 | 0.018311 | -3.13666 |
| REACTOME_ARYL_HYDROCARBON_RECEPTOR_SIGNALLING | -0.47908 | 0.032834 | -3.273 | 0.002236 | -1.44834 |
| KAUFFMANN_MELANOMA_RELAPSE_DN | -0.50714 | -0.04202 | -2.84527 | 0.007042 | -2.37586 |
| HEIDENBLAD_AMPLIFIED_IN_BONE_CANCER | -0.51606 | -0.03704 | -3.72569 | 0.000617 | -0.3977 |
| REACTOME_APEX1_INDEPENDENT_RESOLUTION_OF_AP_SITES_VIA_THE_SINGLE_NUCLEOTIDE_REPLACEMENT_PATHWAY | -0.60479 | 0.073232 | -4.62591 | 4.06E-05 | 1.828355 |

GSVA:Gene Set Variation Analysis.

Supplementary Table S5. GO and KEGG enrichment analysis results.

| ONTOLOGY | ID | Description | BgRatio | pvalue | qvalue | Count |
| --- | --- | --- | --- | --- | --- | --- |
| BP | GO:0045860 | positive regulation of protein kinase activity | 396/18800 | 2.35E-06 | 0.001701 | 9 |
| BP | GO:0033674 | positive regulation of kinase activity | 476/18800 | 1.04E-05 | 0.003772 | 9 |
| BP | GO:0032147 | activation of protein kinase activity | 142/18800 | 3.97E-06 | 0.001915 | 6 |
| BP | GO:0097191 | extrinsic apoptotic signaling pathway | 221/18800 | 4.9E-05 | 0.01417 | 6 |
| BP | GO:0097193 | intrinsic apoptotic signaling pathway | 295/18800 | 0.000238 | 0.04014 | 6 |
| CC | GO:0045121 | membrane raft | 326/19594 | 0.000327 | 0.024988 | 6 |
| CC | GO:0098857 | membrane microdomain | 327/19594 | 0.000332 | 0.024988 | 6 |
| CC | GO:0005874 | microtubule | 435/19594 | 0.00147 | 0.050246 | 6 |
| CC | GO:0009898 | cytoplasmic side of plasma membrane | 169/19594 | 0.001383 | 0.050246 | 4 |
| CC | GO:0098562 | cytoplasmic side of membrane | 193/19594 | 0.002247 | 0.056375 | 4 |
| MF | GO:0005525 | GTP binding | 379/18410 | 0.000997 | 0.06001 | 6 |
| MF | GO:0019001 | guanyl nucleotide binding | 401/18410 | 0.001332 | 0.06001 | 6 |
| MF | GO:0032561 | guanyl ribonucleotide binding | 401/18410 | 0.001332 | 0.06001 | 6 |
| MF | GO:0031625 | ubiquitin protein ligase binding | 298/18410 | 0.002091 | 0.078493 | 5 |
| MF | GO:0050811 | GABA receptor binding | 17/18410 | 0.0012 | 0.06001 | 2 |
| KEGG | hsa04210 | Apoptosis | 136/8164 | 0.000552 | 0.036954 | 5 |
| KEGG | hsa04140 | Autophagy - animal | 141/8164 | 0.00065 | 0.036954 | 5 |
| KEGG | hsa05160 | Hepatitis C | 157/8164 | 0.001057 | 0.036954 | 5 |
| KEGG | hsa05130 | Pathogenic Escherichia coli infection | 197/8164 | 0.002877 | 0.062837 | 5 |
| KEGG | hsa05207 | Chemical carcinogenesis - receptor activation | 212/8164 | 0.003944 | 0.06434 | 5 |

GO:Gene Ontology;BP,biological process;CC:cellular component.

Supplementary Table S6. GSEA enrichment analysis results of Combined dataset Low- High riskscore group genes.

| ID | setSize | enrichmentScore | NES | pvalue | qvalue | rank |
| --- | --- | --- | --- | --- | --- | --- |
| REACTOME_GOLGI_TO_ER_RETROGRADE_TRANSPORT | 103 | 0.598568 | 2.49088 | 0.002141 | 0.028559 | 2215 |
| REACTOME_INTRA_GOLGI_AND_RETROGRADE_GOLGI_TO_ER_TRAFFIC | 155 | 0.494735 | 2.184087 | 0.002105 | 0.028559 | 2230 |
| REACTOME_UNFOLDED_PROTEIN_RESPONSE_UPR | 77 | 0.532883 | 2.105766 | 0.002062 | 0.028559 | 2238 |
| WP_IL18_SIGNALING_PATHWAY | 242 | 0.350383 | 1.641648 | 0.002132 | 0.028559 | 803 |
| WP_PHOTODYNAMIC_THERAPYINDUCED_UNFOLDED_PROTEIN_RESPONSE | 21 | 0.561911 | 1.676376 | 0.010142 | 0.061221 | 2076 |
| PID_IL2_PI3K_PATHWAY | 34 | -0.43355 | -1.42758 | 0.046332 | 0.136809 | 1852 |

GSEA:Gene Set Enrichment Analysis.

Supplementary Table S7. GSVA analysis results of Combined dataset High-Low Riskscore group genes.

|  | logFC | AveExpr | t | P.Value | B |
| --- | --- | --- | --- | --- | --- |
| REACTOME_CREB3_FACTORS_ACTIVATE_GENES | 0.930235 | -0.00451 | 7.247981 | 1.29E-07 | 7.336566 |
| YAMANAKA_GLIOBLASTOMA_SURVIVAL_UP | 0.784776 | 0.062624 | 5.963745 | 3.06E-06 | 4.530366 |
| BIERIE_INFLAMMATORY_RESPONSE_TGFB1 | 0.733392 | 0.042713 | 4.03322 | 0.000449 | 0.018614 |
| REACTOME_ATTACHMENT_OF_GPI_ANCHOR_TO_UPAR | 0.71634 | -0.00077 | 5.901519 | 3.58E-06 | 4.388794 |
| SAMOLS_TARGETS_OF_KHSV_MIRNAS_UP | 0.70366 | 0.052237 | 4.831922 | 5.65E-05 | 1.900276 |
| TOMIDA_LUNG_CANCER_POOR_SURVIVAL | 0.700455 | 0.010479 | 4.152657 | 0.00033 | 0.298405 |
| WANG_ESOPHAGUS_CANCER_PROGRESSION_UP | 0.673326 | 0.054837 | 4.221652 | 0.000276 | 0.460485 |
| TURJANSKI_MAPK11_TARGETS | 0.659255 | 0.030082 | 3.813772 | 0.00079 | -0.49199 |
| JONES_TCOF1_TARGETS | 0.627728 | -0.01186 | 3.495771 | 0.00177 | -1.22042 |
| NIKOLSKY_BREAST_CANCER_1Q32_AMPLICON | 0.62459 | 0.005023 | 4.074504 | 0.000404 | 0.115199 |
| TONKS_TARGETS_OF_RUNX1_RUNX1T1_FUSION_SUSTAINED_IN_GRANULOCYTE_DN | -0.64919 | -0.04352 | -5.68128 | 6.29E-06 | 3.884271 |
| WP_EICOSANOID_METABOLISM_VIA_CYTOCHROME_P450_MONOOXYGENASES_PATHWAY | -0.65222 | -0.05486 | -4.21183 | 0.000283 | 0.437387 |
| REACTOME_SEROTONIN_AND_MELATONIN_BIOSYNTHESIS | -0.65866 | -0.07166 | -3.50868 | 0.001713 | -1.19118 |
| LOPEZ_MESOTHELIOMA_SURVIVAL_WORST_VS_BEST_DN | -0.6703 | -0.07299 | -4.04323 | 0.000438 | 0.042027 |
| BIOCARTA_PELP1_PATHWAY | -0.70269 | -0.00538 | -5.9145 | 3.47E-06 | 4.41836 |
| REACTOME_CONJUGATION_OF_BENZOATE_WITH_GLYCINE | -0.72058 | 0.00067 | -3.35478 | 0.002518 | -1.53755 |
| TESAR_ALK_AND_JAK_TARGETS_MOUSE_ES_D4_DN | -0.77368 | -0.01119 | -3.00909 | 0.005876 | -2.29482 |
| REACTOME_AMINO_ACID_CONJUGATION | -0.77402 | -0.09284 | -3.87122 | 0.000682 | -0.35882 |
| SCHLESINGER_METHYLATED_IN_COLON_CANCER | -0.78618 | 0.014146 | -5.51463 | 9.64E-06 | 3.499249 |
| REACTOME_FRUCTOSE_CATABOLISM | -0.8659 | 0.039241 | -5.49281 | 1.02E-05 | 3.448656 |

GSVA,Gene Set Variation Analysis.

Supplementary Table S8. mRNA-TF interaction network nodes.

| mRNA | TF |  | mRNA | TF |  | mRNA | TF |
| --- | --- | --- | --- | --- | --- | --- | --- |
| CYB5R4 | BHLHE40 |  | C1QBP | HNF4A |  | RAB1B | SP1 |
| CYB5R4 | CEBPB |  | C1QBP | KMT2A |  | RAB1B | SPI1 |
| CYB5R4 | CTCF |  | C1QBP | MAX |  | RAB1B | TBP |
| CYB5R4 | E2F1 |  | C1QBP | MAZ |  | RAB1B | YY1 |
| CYB5R4 | E2F6 |  | C1QBP | MXI1 |  | RNF13 | ELF1 |
| CYB5R4 | ELF1 |  | C1QBP | MYC |  | RNF13 | GABPA |
| CYB5R4 | ELK1 |  | C1QBP | MYCN |  | RNF13 | MAX |
| CYB5R4 | ERG |  | C1QBP | NELFA |  | RNF13 | MYC |
| CYB5R4 | ETS1 |  | C1QBP | POLR2A |  | RNF13 | NELFA |
| CYB5R4 | ETV1 |  | C1QBP | RAD21 |  | RNF13 | NFYA |
| CYB5R4 | FOXA1 |  | C1QBP | SMC3 |  | RNF13 | POLR2A |
| CYB5R4 | GABPA |  | C1QBP | SPI1 |  | RNF13 | SP1 |
| CYB5R4 | HNF4A |  | C1QBP | STAG1 |  | RNF13 | SP2 |
| CYB5R4 | KMT2A |  | C1QBP | STAT3 |  | RNF13 | SPI1 |
| CYB5R4 | MAX |  | C1QBP | TBP |  | RNF13 | TBP |
| CYB5R4 | MAZ |  | C1QBP | TEAD1 |  | RNF13 | TFAP2A |
| CYB5R4 | MXI1 |  | C1QBP | USF1 |  | RNF13 | TFAP2C |
| CYB5R4 | MYC |  | C1QBP | USF2 |  | SERP1 | E2F1 |
| CYB5R4 | MYCN |  | C1QBP | VDR |  | SERP1 | ELF1 |
| CYB5R4 | NELFA |  | C1QBP | ZNF384 |  | SERP1 | ERG |
| CYB5R4 | POLR2A |  | CES2 | ELF1 |  | SERP1 | ETS1 |
| CYB5R4 | RAD21 |  | CES2 | ERG |  | SERP1 | GABPA |
| CYB5R4 | SMC3 |  | CES2 | ETS1 |  | SERP1 | KMT2A |
| CYB5R4 | SPI1 |  | CES2 | ETV1 |  | SERP1 | MAZ |
| CYB5R4 | STAG1 |  | CES2 | FLI1 |  | SERP1 | NELFA |
| CYB5R4 | STAT3 |  | CES2 | FOS |  | SERP1 | NRF1 |
| CYB5R4 | TBP |  | CES2 | GABPA |  | SERP1 | POLR2A |
| CYB5R4 | TEAD1 |  | CES2 | NELFA |  | SERP1 | SPI1 |
| CYB5R4 | USF1 |  | CES2 | NRF1 |  | SERP1 | SUPT5H |
| CYB5R4 | USF2 |  | CES2 | POLR2A |  | SERP1 | TBP |
| CYB5R4 | VDR |  | CES2 | RUNX1 |  | SERP1 | YY1 |
| CYB5R4 | ZNF384 |  | CES2 | SPI1 |  | SERP1 | CEBPA |
| C1QBP | BHLHE40 |  | CES2 | SUPT5H |  | SERP1 | CEBPB |
| C1QBP | CEBPB |  | CYB5R4 | NRF1 |  | SERP1 | CREB1 |
| C1QBP | CTCF |  | CYB5R4 | REST |  | UFSP2 | FOS |
| C1QBP | E2F1 |  | RAB1B | CTCF |  | UFSP2 | FOXA1 |
| C1QBP | E2F6 |  | RAB1B | CTCFL |  | UFSP2 | ATF1 |
| C1QBP | ELF1 |  | RAB1B | E2F1 |  | UFSP2 | ATF2 |
| C1QBP | ELK1 |  | RAB1B | EGR1 |  | UFSP2 | HOXB13 |
| C1QBP | ERG |  | RAB1B | ELF1 |  | UFSP2 | NRF1 |
| C1QBP | ETS1 |  | RAB1B | ESRRA |  | UFSP2 | SPI1 |
| C1QBP | ETV1 |  | RAB1B | ETV1 |  | UFSP2 | YY1 |
| C1QBP | FOXA1 |  | RAB1B | GABPA |  | UFSP2 | CREB1 |
| C1QBP | GABPA |  | RAB1B | NRF1 |  |  |  |

TF:Transcription factors.

Supplementary Table S9. mRNA-drug interaction network nodes.

| mRNA | drug |  | mRNA | drug |
| --- | --- | --- | --- | --- |
| CYB5R4 | Tretinoin |  | CES2 | Methylphenidate |
| CYB5R4 | Valproic Acid | | CES2 | Paraoxon |
| CES2 | 4-nitrophenyl acetate | | CES2 | Permethrin |
| CES2 | 6-O-monoacetylmorphine | | CES2 | Phenobarbital |
| CES2 | Acetaminophen | | CES2 | Procaine |
| CES2 | Benzo(a)pyrene | | CES2 | temocapril hydrochloride |
| CES2 | bioresmethrin | | CES2 | Trinitrobenzenesulfonic Acid |
| CES2 | Cholesterol | | RAB1B | Valproic Acid |
| CES2 | cocaethylene | | RNF13 | Valproic Acid |
| CES2 | Cocaine |  | SERP1 | Cyclosporine |
| CES2 | Cyclosporine | | SERP1 | sodium arsenite |
| CES2 | Ethanol |  | SERP1 | Thapsigargin |
| CES2 | Fluorouracil | | SERP1 | Tobacco Smoke Pollution |
| CES2 | Heroin |  | SERP1 | Tunicamycin |
| CES2 | Irinotecan |  | SERP1 | Valproic Acid |
| CES2 | Loperamide | |  |  |

Supplementary Table 10. mRNA-miRNA interaction network nodes.

| miRNA | mRNA |  | miRNA | mRNA |
| --- | --- | --- | --- | --- |
| hsa-miR-24-3p | CES2 |  | hsa-miR-1-3p | SERP1 |
| hsa-miR-4726-5p | CES2 |  | hsa-miR-125b-5p | SERP1 |
| hsa-miR-130a-3p | CYB5R4 |  | hsa-miR-132-3p | SERP1 |
| hsa-miR-361-5p | CYB5R4 |  | hsa-miR-137 | SERP1 |
| hsa-miR-181c-5p | RAB1B |  | hsa-miR-145-5p | SERP1 |
| hsa-miR-214-3p | RAB1B |  | hsa-miR-125a-5p | SERP1 |
| hsa-miR-195-5p | RAB1B |  | hsa-miR-376c-3p | SERP1 |
| hsa-miR-135b-5p | RAB1B |  | hsa-miR-370-3p | SERP1 |
| hsa-miR-181d-5p | RAB1B |  | hsa-miR-380-3p | SERP1 |
| hsa-miR-455-5p | RAB1B |  | hsa-miR-381-3p | SERP1 |
| hsa-miR-769-5p | RAB1B |  | hsa-miR-433-3p | SERP1 |
| hsa-let-7d-5p | RNF13 |  | hsa-miR-409-5p | SERP1 |
| hsa-miR-20a-5p | RNF13 |  | hsa-miR-410-3p | SERP1 |
| hsa-miR-25-3p | RNF13 |  | hsa-miR-493-5p | SERP1 |
| hsa-miR-32-5p | RNF13 |  | hsa-miR-432-5p | SERP1 |
| hsa-miR-92a-3p | RNF13 |  | hsa-miR-494-3p | SERP1 |
| hsa-miR-107 | RNF13 |  | hsa-miR-495-3p | SERP1 |
| hsa-miR-106b-5p | RNF13 |  | hsa-miR-181d-5p | SERP1 |
| hsa-miR-376c-3p | RNF13 |  | hsa-miR-655-3p | SERP1 |
| hsa-miR-493-5p | RNF13 |  | hsa-miR-140-3p | SERP1 |
| hsa-miR-181a-5p | SERP1 |  | hsa-miR-411-3p | SERP1 |
| hsa-miR-212-3p | SERP1 |  | hsa-miR-543 | SERP1 |
| hsa-miR-218-5p | SERP1 |  |  |  |

Supplementary Table S11. mRNA-RBP interaction network nodes.

| mRNA | RBP |  | mRNA | RBP |  | mRNA | RBP |
| --- | --- | --- | --- | --- | --- | --- | --- |
| C1QBP | ALYREF |  | CYB5R4 | HNRNPC |  | RNF13 | ELAVL1 |
| C1QBP | DDX3X |  | CYB5R4 | IGF2BP2 |  | RNF13 | ELAVL3 |
| C1QBP | ELAVL1 |  | CYB5R4 | IGF2BP3 |  | RNF13 | FUS |
| C1QBP | ELAVL3 |  | CYB5R4 | RBM4 |  | RNF13 | HNRNPC |
| C1QBP | FUS |  | CYB5R4 | RBMX |  | RNF13 | PTBP1 |
| C1QBP | G3BP1 |  | CYB5R4 | TARDBP |  | RNF13 | RBFOX2 |
| C1QBP | HNRNPC |  | RAB1B | ALYREF |  | RNF13 | RBMX |
| C1QBP | IGF2BP1 |  | RAB1B | DDX3X |  | RNF13 | TARDBP |
| C1QBP | IGF2BP3 |  | RAB1B | ELAVL1 |  | RNF13 | U2AF2 |
| C1QBP | LIN28B |  | RAB1B | G3BP1 |  | SERP1 | DDX3X |
| C1QBP | RBFOX2 |  | RAB1B | HNRNPC |  | SERP1 | ELAVL1 |
| C1QBP | RBMX |  | RAB1B | HNRNPK |  | SERP1 | ELAVL3 |
| C1QBP | RNPS1 |  | RAB1B | IGF2BP1 |  | SERP1 | FUS |
| C1QBP | SCAF4 |  | RAB1B | IGF2BP2 |  | SERP1 | G3BP1 |
| C1QBP | SCAF8 |  | RAB1B | IGF2BP3 |  | SERP1 | HNRNPC |
| C1QBP | TARDBP |  | RAB1B | LIN28B |  | SERP1 | HNRNPL |
| C1QBP | TIA1 |  | RAB1B | METTL1 |  | SERP1 | IGF2BP1 |
| C1QBP | U2AF2 |  | RAB1B | PCBP2 |  | SERP1 | IGF2BP3 |
| C1QBP | UPF1 |  | RAB1B | RBFOX2 |  | SERP1 | PTBP1 |
| C1QBP | YBX1 |  | RAB1B | RBMX |  | SERP1 | RBMX |
| C1QBP | YTHDC1 |  | RAB1B | RNPS1 |  | SERP1 | RNPS1 |
| C1QBP | YTHDF1 |  | RAB1B | SCAF4 |  | SERP1 | SCAF4 |
| C1QBP | YTHDF3 |  | RAB1B | SCAF8 |  | SERP1 | SCAF8 |
| CES2 | ALYREF |  | RAB1B | TARDBP |  | SERP1 | TARDBP |
| CES2 | DDX3X |  | RAB1B | U2AF1 |  | SERP1 | U2AF1 |
| CES2 | RBFOX2 |  | RAB1B | U2AF2 |  | SERP1 | U2AF2 |
| CES2 | RBMX |  | RAB1B | UPF1 |  | SERP1 | UPF1 |
| CES2 | SCAF4 |  | RAB1B | YBX1 |  | SERP1 | YTHDC1 |
| CES2 | UPF1 |  | RAB1B | YTHDC1 |  | SERP1 | YTHDF1 |
| CES2 | YTHDC1 |  | RNF13 | ALYREF |  | UFSP2 | ELAVL1 |
| CES2 | YTHDF1 |  | RNF13 | DDX3X |  | UFSP2 | SCAF4 |
| CYB5R4 | DDX3X |  | RNF13 | DHX9 |  | UFSP2 | TARDBP |
| CYB5R4 | ELAVL1 |  |  |  |  |  |  |

RBP,RNA binding protein.
