# Supplementary figures and images for: Identification of Endoplasmic Reticulum Stress-Related Genes in Osteoporosis Pathogenesis
Source: Mediators Inflamm. 2025 Aug 30;2025:6726771. doi: 10.1155/mi/6726771 (PMC12413945; doi:10.1155/mi/6726771)

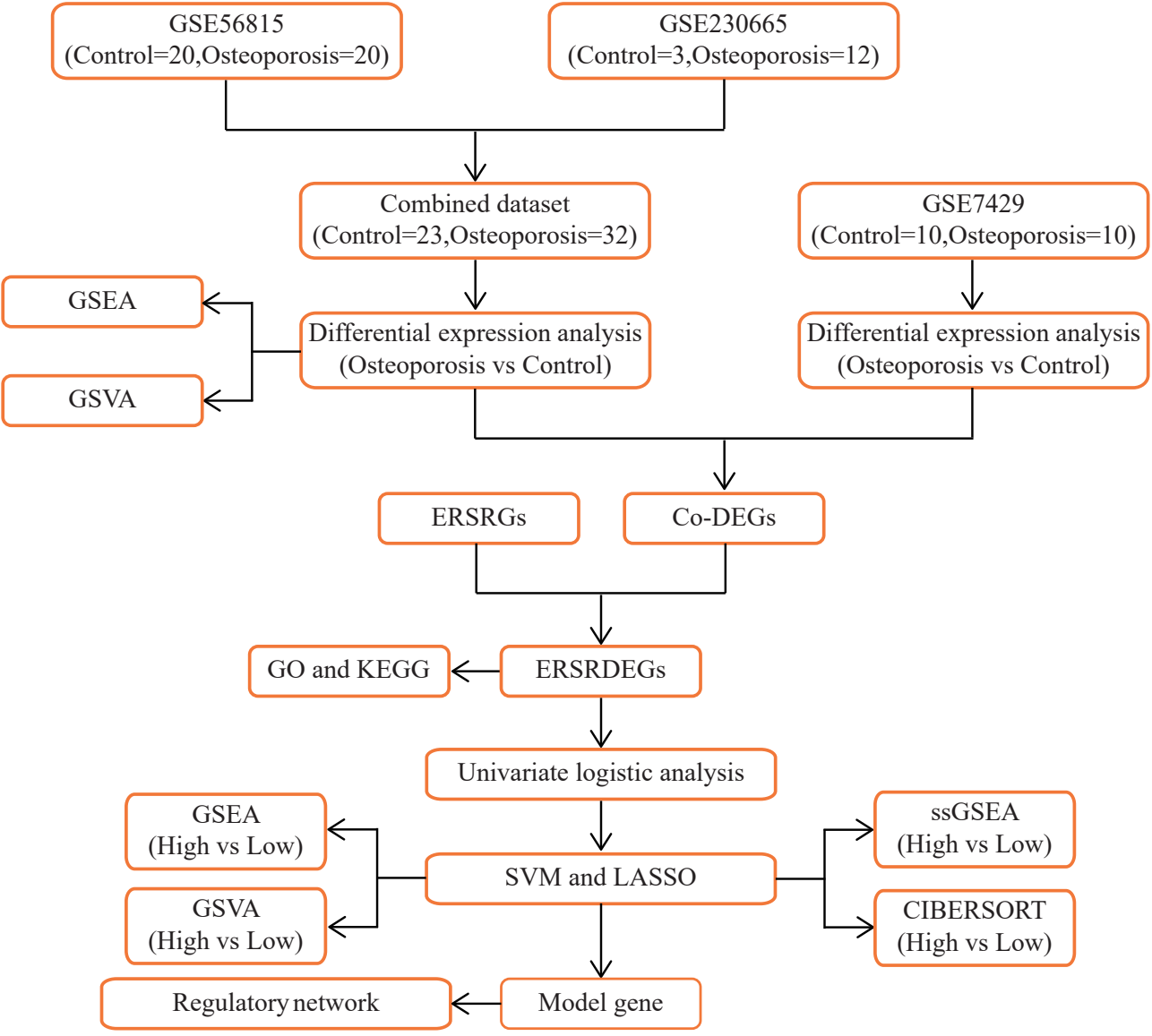

Supplement: Supporting Information 2 — Figure S1: GSEA, gene set enrichment analysis; GSVA, gene set variation analysis; Co-DEGs, common differentially expressed genes; ERSRGs, endoplasmic reticulum stress related genes; ERSRDEGs, endoplasmic reticulum stress related differentially expressed genes; GO, Gene Ontology; KEGG, Kyoto Encyclopedia of Genes and Genomes; SVM, support vector machine; LASSO, least absolute shrinkage and selection operator; ssGSEA, single-sample gene-set enrichment analysis. [file 6726771.f2.pdf]

A

GSE7429 Before

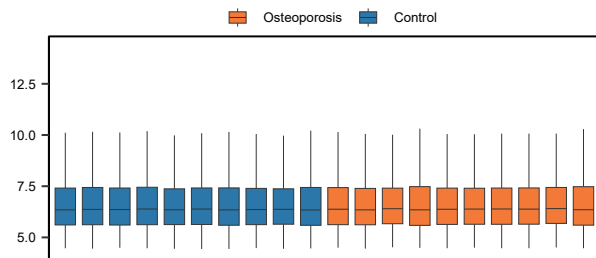

B

GSE7429 After

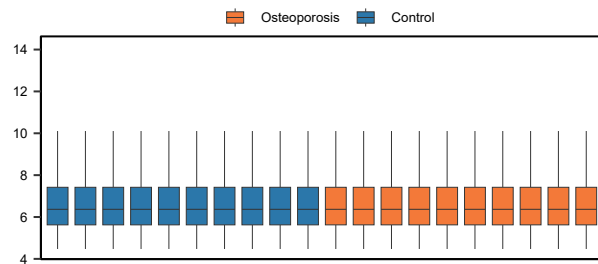

C

Before

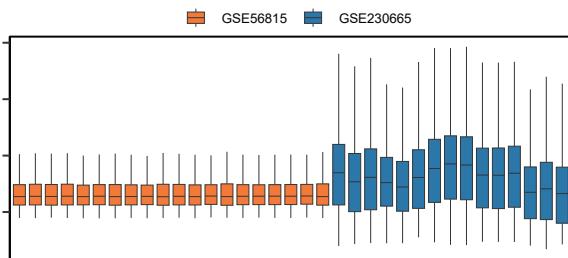

D

After

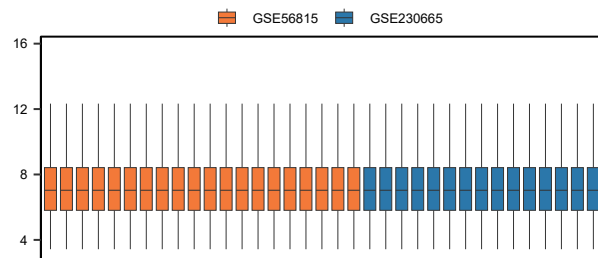

E

Before

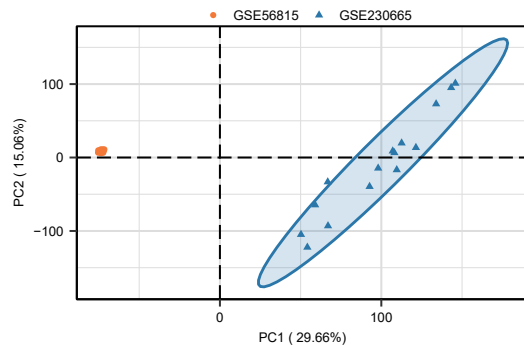

F

After

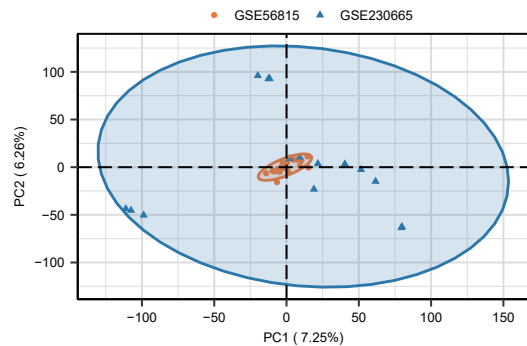

Supplement: Supporting Information 3 — Figure S2: (A) Box plot of GSE7429 sample before normalization. (B) Box plot of GSE7429 sample after normalization. (C) Box plot of the combined dataset samples before standardization. (D) Box plot of the standardized combined dataset samples. (E) PCA plot of the combined dataset before batch processing. (F) PCA plot of the combined dataset after debatch processing. PCA, principal component analysis. [file 6726771.f3.pdf]

A

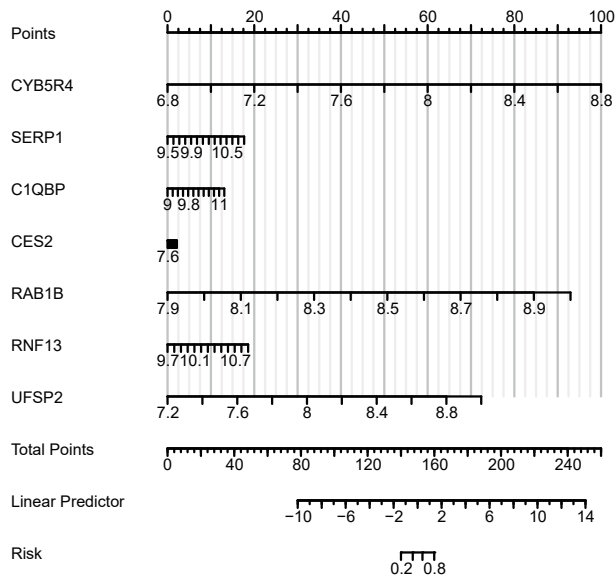

B

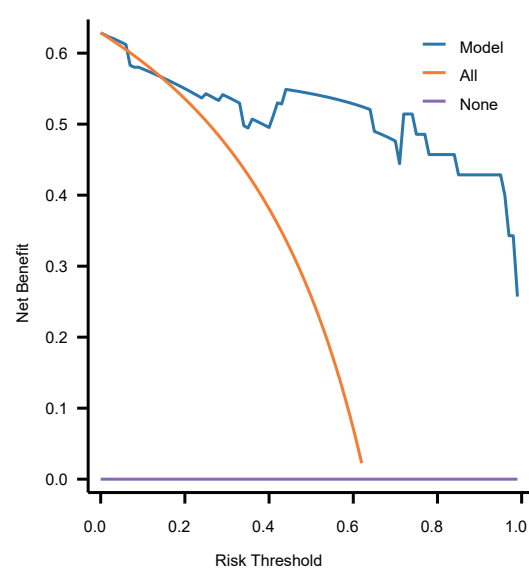

C

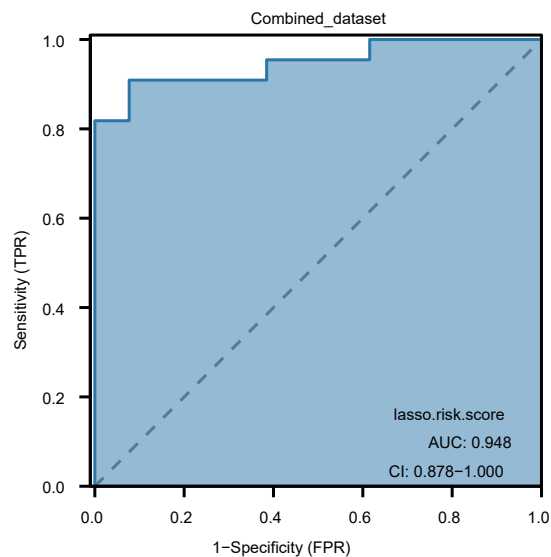

D

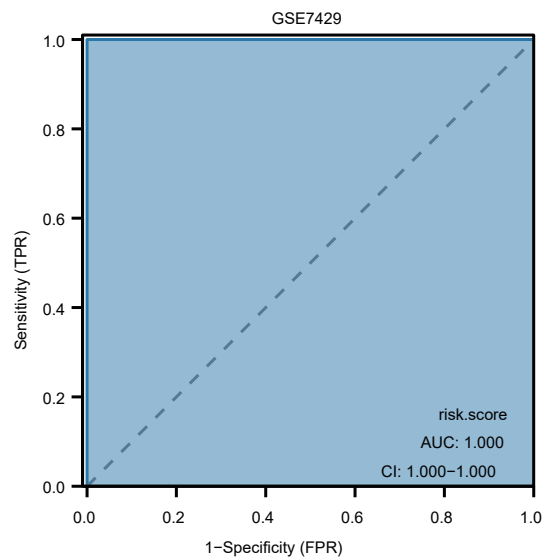

Supplement: Supporting Information 5 — Figure S4: (A) Nomogram of model genes in the osteoporosis diagnostic model based on the combined dataset. (B) DCA diagram of the osteoporosis diagnostic model based on model genes in the combined GEO dataset. (C) ROC curve of the risk score of the com-bined dataset osteoporosis diagnostic model. (D) ROC curve of the risk score of the GSE7429 osteoporosis diagnostic model. DCA, decision curve analysis; ROC, receiver operating characteristic; AUC, area under the curve. The model has higher accuracy when AUC is above 0.9, the model has certain accuracy when AUC is between 0.7 and 0.9, and the model has lower accuracy when AUC is between 0.5 and 0.7. [file 6726771.f5.pdf]

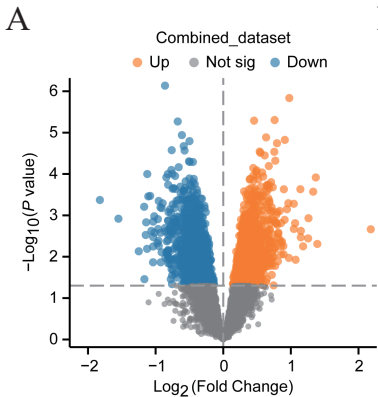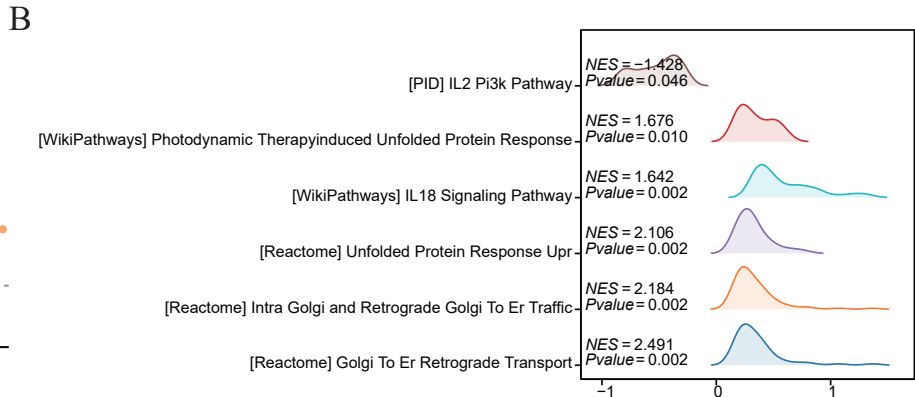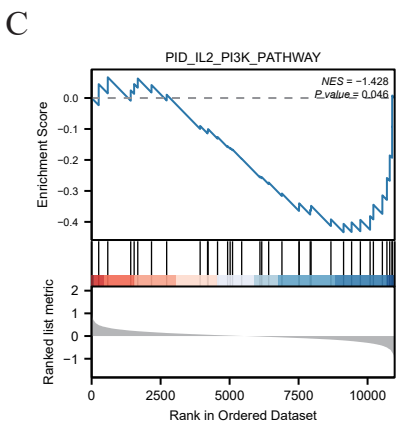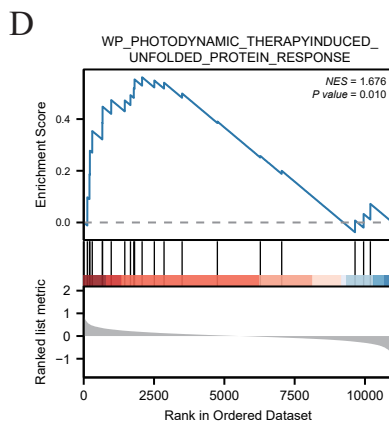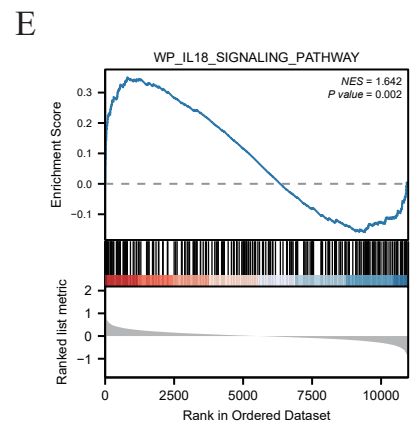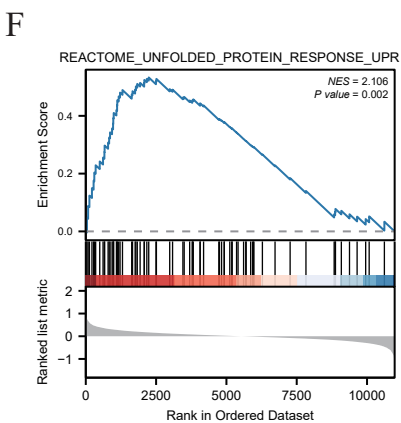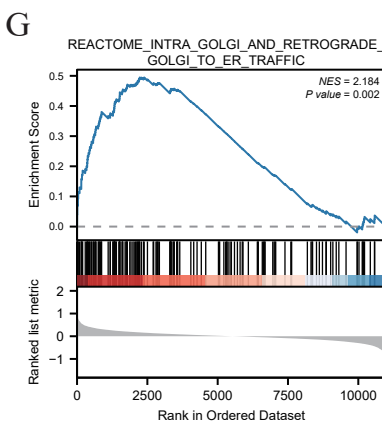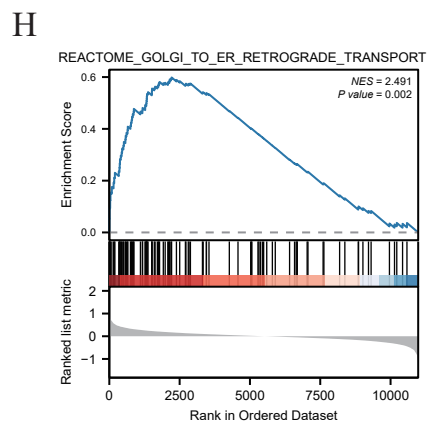

Supplement: Supporting Information 6 — Figure S5: (A) A volcano plot showing the results of differential gene expression analysis between high- and low-risk groups in the combined GEO dataset. (B) GSEA of the combined dataset shows an enrichment plot of six biological functions. (C–H) GSEA shows that ERSRDEGs are significantly enriched in: IL2-PI3K pathway (C), photodynamic therapy-induced UPR (D), IL-18 signaling pathway (E), unfolded protein response (F), intra-golgi and retrograde golgi-to-ER traffic (G), and golgi-to-ER retrograde transport (H). ERSRDEGs, endoplasmic reticulum stress-related differentially expressed genes; GSEA, gene set enrichment analysis. The screening criterion for GSEA is p < 0.05. [file 6726771.f6.pdf]

A

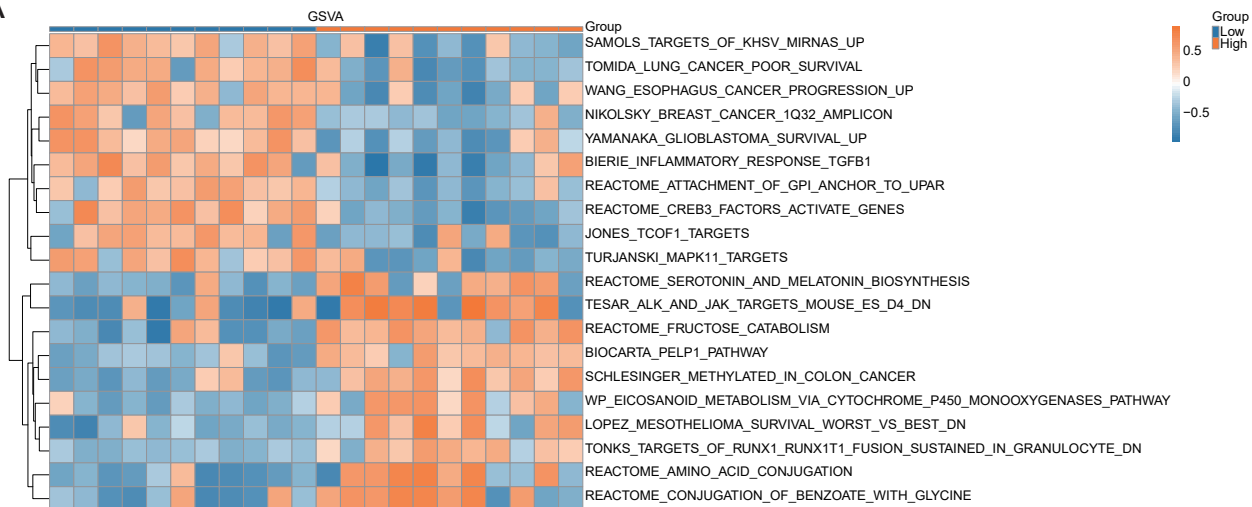

B

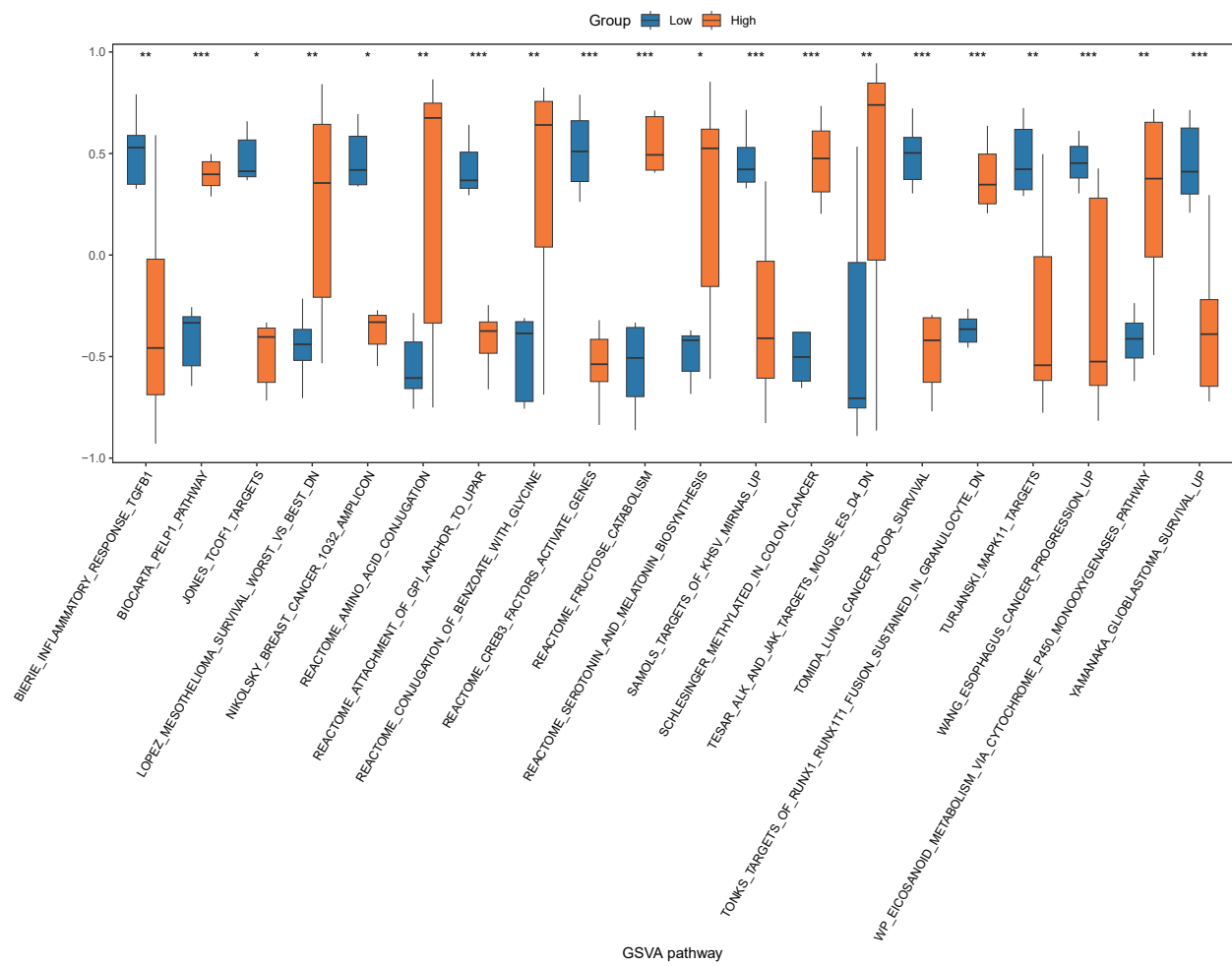

Supplement: Supporting Information 7 — Figure S6: (A) Group comparison diagram and (B) heat map of GSVA results between high-risk and low-risk group samples in the combined dataset. GSVA, gene set variation analysis. ⁣∗ represents p < 0.05; ⁣∗∗ represents p < 0.01, which is highly statistically significant; ⁣∗∗∗ represents p < 0.001, which is extremely statistically significant. GSVA screening was performed using p < 0.05 and log FC ranking to identify the top 10 positively and negatively enriched pathways. In the heat map, blue represents the low-risk group samples, while orange represents the high-risk group samples in the combined dataset. [file 6726771.f7.pdf]

A

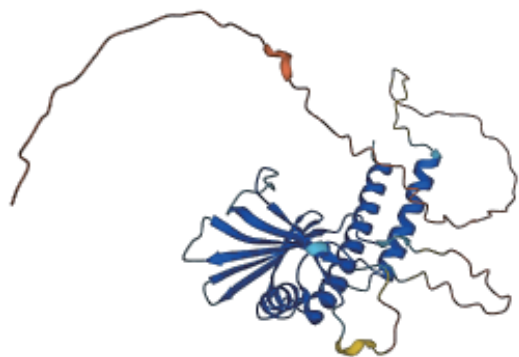

C1QBP

B

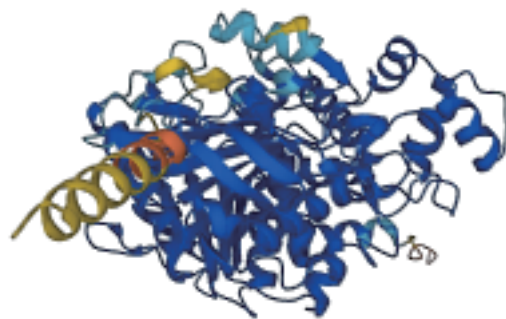

CES2

C

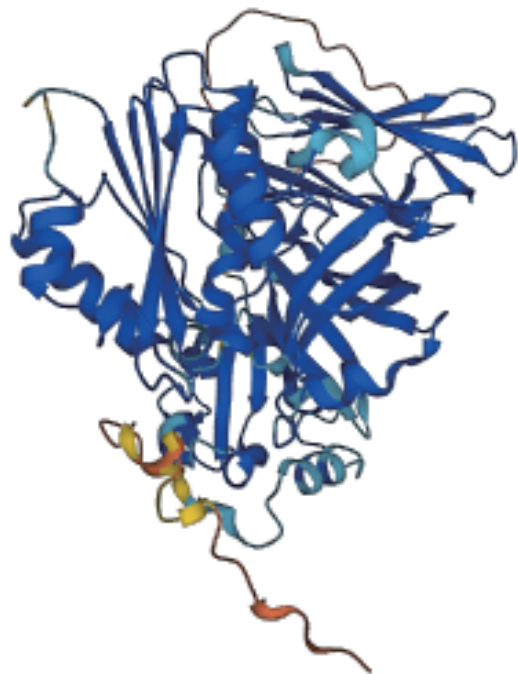

CYB5R4

D

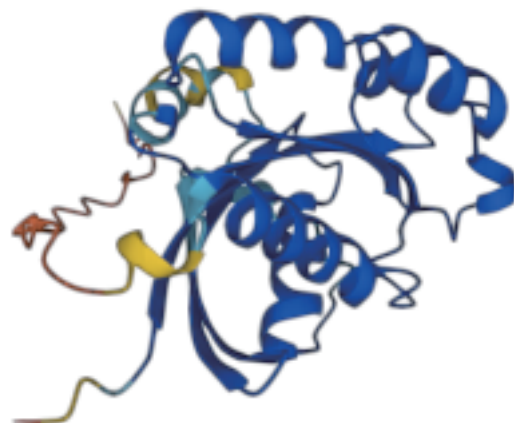

RAB1B

E

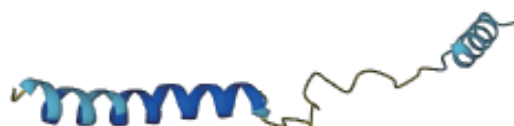

SERP1

G

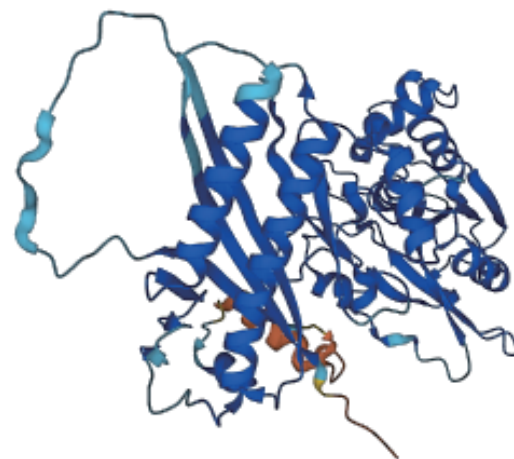

UFSP2

F

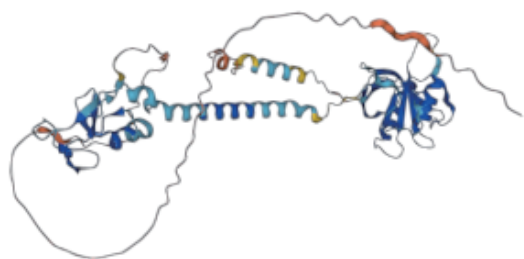

RNF13

Supplement: Supporting Information 8 — Figure S7: (A) C1QBP, (B) CES2, (C) CYB5R4, (D) RAB1B, (E) SERP1, (F) RNF13, and (G) UFSP2. The AlphaFold website generates per residue confidence scores (pLDDT) ranging from 0 to 100. Regions with pLDDT scores below 50 might represent isolated un-structured areas. When pLDDT < 50 (red regions), the model confidence is very low; when 50 < pLDDT < 70 (yellow regions), the model confidence is low; when 70 < pLDDT < 90 (light blue regions), the model confidence is moderate; when pLDDT > 90 (blue regions), the model confidence is very high. [file 6726771.f8.pdf]
