# Supplementary material for: Identification of Endoplasmic Reticulum Stress-Related Genes in Osteoporosis Pathogenesis
Source: Mediators Inflamm. 2025 Aug 30;2025:6726771. doi: 10.1155/mi/6726771 (PMC12413945; doi:10.1155/mi/6726771)
Supplement: Supporting Information 4 — Figure S3: (A–E) KEGG enrichment analysis results of ERSRDEGs. Visual display of pathway diagram: Chemical carcinogene-sis-receptor activation (A), pathogenic Escherichia coli infection (B), hepatitis C (C), autophagy-animal (D), and apoptosis (E). KEGG, Kyoto Encyclopedia of Genes and Genomes; ERSRDEGs, endoplasmic reticulum stress related differentially expressed genes. [file 6726771.f4.pdf]

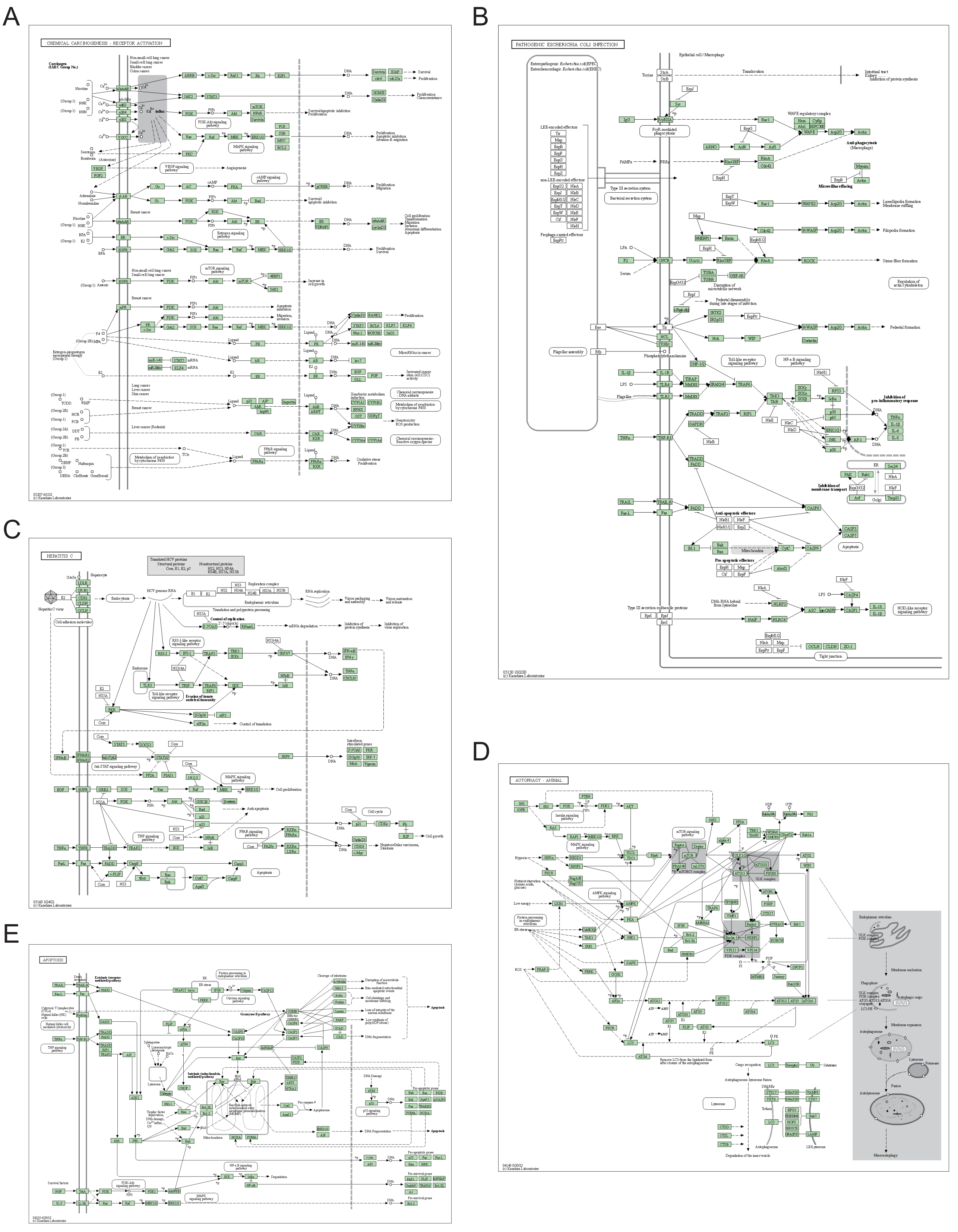
A
CHEMICAL CARCINOGENESIS - RECEPTOR ACTIVATION
B
PATHOGENIC ESCHERICHIA COLI INFECTION
C
HEPATITIS C
D
AUTOPHAGY - ANIMAL
E
APOPTOSIS
